# Ectopic callose deposition into woody biomass modulates the nano-architecture of macrofibrils

**DOI:** 10.1038/s41477-023-01459-0

**Published:** 2023-09-04

**Authors:** Matthieu Bourdon, Jan J. Lyczakowski, Rosalie Cresswell, Sam Amsbury, Francisco Vilaplana, Marie-Joo Le Guen, Nadège Follain, Raymond Wightman, Chang Su, Fulgencio Alatorre-Cobos, Maximilian Ritter, Aleksandra Liszka, Oliver M. Terrett, Shri Ram Yadav, Anne Vatén, Kaisa Nieminen, Gugan Eswaran, Juan Alonso-Serra, Karin H. Müller, Dinu Iuga, Pal Csaba Miskolczi, Lothar Kalmbach, Sofia Otero, Ari Pekka Mähönen, Rishikesh Bhalerao, Vincent Bulone, Shawn D. Mansfield, Stefan Hill, Ingo Burgert, Johnny Beaugrand, Yoselin Benitez-Alfonso, Ray Dupree, Paul Dupree, Ykä Helariutta

**Affiliations:** 1 The Sainsbury Laboratory, University of Cambridge, Cambridge, UK; 2 Department of Biochemistry, University of Cambridge, Cambridge, UK; 3 Department of Plant Biotechnology, Faculty of Biochemistry, Biophysics and Biotechnology, Jagiellonian University, Krakow, Poland; 4 Department of Physics, University of Warwick, Coventry, UK; 5 Centre for Plant Science, Faculty of Biological Sciences, University of Leeds, Leeds, UK; 6 Division of Glycoscience, Department of Chemistry, KTH Royal Institute of Technology, Stockholm, Sweden; 7 Wallenberg Wood Science Centre (WWSC), KTH Royal Institute of Technology, Stockholm, Sweden; 8 Scion, Te Papa Tipu Innovation Park, Rotorua, New Zealand; 9 Normandie Université, UNIROUEN Normandie, INSA Rouen, CNRS, PBS, Rouen, France; 10 Wood Development Group, University of Helsinki, Helsinki, Finland; 11 Wood Materials Science, Institute for Building Materials, ETH Zürich, Zürich, Switzerland; 12 Empa Wood Tec, Cellulose and Wood Materials Laboratory, Dübendorf, Switzerland; 13 Doctoral School of Exact and Natural Sciences, Jagiellonian University, Krakow, Poland; 14 Organismal and Evolutionary Biology Research Programme, Faculty of Biological and Environmental Sciences and Viikki Plant Science Centre, University of Helsinki, Helsinki, Finland; 15 Production systems / Tree Breeding Department, Natural Resources Institute Finland (Luke), Helsinki, Finland; 16 Cambridge Advanced Imaging Centre, Department of Physiology, Development and Neuroscience, Cambridge, UK; 17 Umeå Plant Science Centre, Department of Forest Genetics and Plant Physiology, Swedish University of Agricultural Sciences, Umeå, Sweden; 18 College of Medicine and Public Health, Flinders University, Bedford Park, South Australia Australia; 19 Department of Wood Science, Faculty of Forestry, University of British Columbia, Vancouver, British Columbia Canada; 20 Department of Botany, University of British Columbia, Vancouver, British Columbia Canada; 21 Biopolymères Interactions Assemblages (BIA), INRA, Nantes, France; 22 The Centre for Plant Science, The Bragg Centre, The Astbury Centre, University of Leeds, Leeds, UK; 23 Present Address: Friedrich Miescher Institute for Biomedical Research (FMI), Basel, Switzerland; 24 Present Address: Plants, Photosynthesis and Soil, School of Biosciences, The University of Sheffield, Sheffield, UK; 25 Present Address: Conacyt-Unidad de Bioquimica y Biologia Molecular de Plantas, Centro de Investigación Científica de Yucatán, Mérida, Mexico; 26 Present Address: Department of Biosciences and Bioengineering, Indian Institute of Technology Roorkee, Roorkee, Uttarakhand India; 27 Present Address: Stomatal Development and Plasticity group, University of Helsinki, Helsinki, Finland; 28 Present Address: UMR 5667 Reproduction et Développement Des Plantes, ENS de Lyon, France; 29 Present Address: Molecular Plant Physiology, Institute of Biology II, University of Freiburg, Freiburg, Germany; 30 Present Address: Science and Technology Office of the Congress of Deputies, Madrid, Spain

**Keywords:** Molecular engineering in plants, Biofuels

## Abstract

Plant biomass plays an increasingly important role in the circular bioeconomy, replacing non-renewable fossil resources. Genetic engineering of this lignocellulosic biomass could benefit biorefinery transformation chains by lowering economic and technological barriers to industrial processing. However, previous efforts have mostly targeted the major constituents of woody biomass: cellulose, hemicellulose and lignin. Here we report the engineering of wood structure through the introduction of callose, a polysaccharide novel to most secondary cell walls. Our multiscale analysis of genetically engineered poplar trees shows that callose deposition modulates cell wall porosity, water and lignin contents and increases the lignin–cellulose distance, ultimately resulting in substantially decreased biomass recalcitrance. We provide a model of the wood cell wall nano-architecture engineered to accommodate the hydrated callose inclusions. Ectopic polymer introduction into biomass manifests in new physico-chemical properties and offers new avenues when considering lignocellulose engineering.

## Abstract (web summary)

Bourdon et al. demonstrate the possibility to ectopically synthesize callose, a polymer restricted to primary cell walls, into *Arabidopsis* and aspen secondary cell walls to manipulate their ultrastructure and ultimately reduce their recalcitrance.

## Main

Fossil fuels have been the major source of energy, chemicals and materials for mankind since the industrial revolution and are concomitantly responsible for a significant portion of greenhouse gas emissions^1^. Confronting this issue requires a substantial change in the production, management and consumption of energy and petroleum-derived products to move towards more sustainable carbon-neutral models. Lignocellulosic biomass (LB) is a promising renewable feedstock for the production of fuels, chemicals and materials. Much LB research has focused on the production of biofuels. There is a need to diversify LB outputs towards the production of specialty chemicals and innovative biomaterials to displace petrochemical outputs^2^ and achieve the carbon-neutral objectives expected in many large-scale production sectors (for example, building, automotive, electronics)^3–6^. To meet this challenge, genetic engineering of LB has been proposed as an upstream solution to directly pre-functionalize its attributes, enabling deconstruction and reassembly for advanced biomaterials applications^2,7,8^.

The bulk of LB is composed of secondary cell walls, which are principally made of cellulose, hemicellulosic polysaccharides and lignin^9,10^. Earlier attempts to genetically engineer LB for specific end uses have logically targeted quantitatively or qualitatively those core polymers^2,7^. Here we report a different approach to lignocellulosic genetic engineering, by introducing an additional polymer (callose) into the intricate nano-architecture of secondary cell wall polymers. Callose is a linear (1,3)-β-glucan and occurs only in specialized plant tissues or domains of the cell wall and/or in response to biotic and abiotic stressors^11^. Linear (1,3)-β-glucans form triple helical conformations in vitro and can assemble into hydrated microfibrillar structures^12–18^. Although callose is not found in most secondary cell walls of woody plants, it has been suggested to contribute to cell wall mechanical properties in compression wood tracheids of several gymnosperm plant species^19–21^ and has been shown to possess load bearing properties in association with cellulose during the dynamic process of pollen tube growth^22^. In addition, it was recently demonstrated that integration of purified (1,3)-β-glucan to cellulose hydrogels can modulate their mechanical properties^23^.

Considering the conformational features and hydration properties of (1,3)-β-glucans, along with their potential involvement in regulating cell wall mechanics, we wanted to assess whether ectopic callose synthesis into the LB of eudicots, the dominant group of plant species, could lead to the acquisition of physico-chemical features of interest for the production of biomass-derived products. To this end, we exploited a previously engineered *Arabidopsis* callose synthase isoform (CALS3) with several gain-of-function mutations that confer enhanced callose biosynthesis in cell walls^24^. Here we used this genetic tool (named *cals3m*) to direct and increase callose content specifically in xylem and fibre cell types of *Arabidopsis thaliana* and hybrid aspen (*Populus tremula* × *tremuloides)* models. We demonstrate that eudicot woody tissues are permissive to integration of an external polymer, callose, allowing us to determine the spatial conformation of callose *in muro*. We show that callose integration leads to a combinatorial effect on lignin content, wood porosity and hygroscopic properties leading to reduced recalcitrance to enzymatic hydrolysis. These findings provide a new strategy to consider when engineering LB for tailored applications and potential to accelerate the implementation of biorefinery processes relying on biomass deconstruction or delignification pipelines such as for biofuels, nanocelluloses and delignified wood.

## Results

### Secondary cell walls can be engineered to contain callose

**Extended Data Fig. 1:**
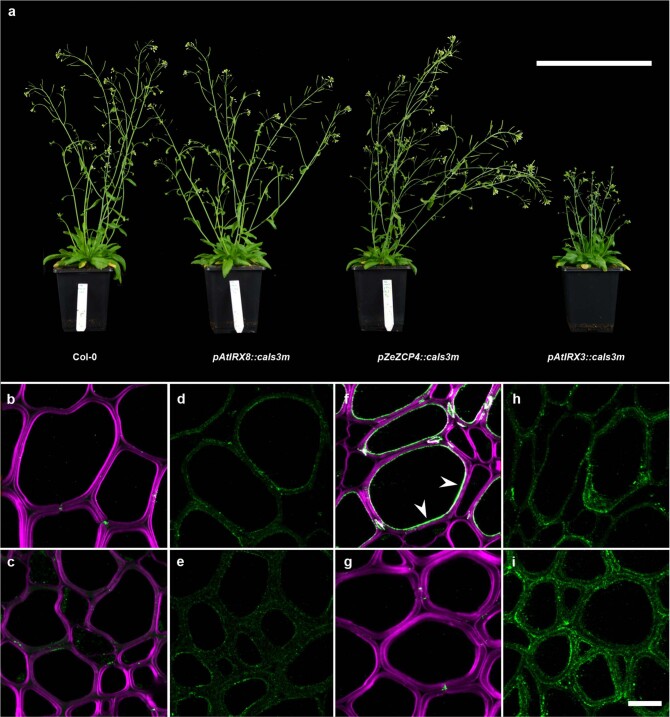
Callose can be effectively and stably integrated in Arabidopsis woody biomass using different secondary cell wall promoters. **a** Representative pictures of *Arabidopsis* WT (Col. 0), *pAtIRX8::cals3m*, *pZeZCP4*::*cals3m* and *pAtIRX3*::*cals3m* individuals after 6 weeks growth in growth chamber conditions (3 independent lines for each construct, n = 8), and their associated callose immunolocalizations (green) after 10 weeks growth, showing callose deposition in xylem vessels (**b**, **d**, **f**, **h**) and interfascicular fibers (**c**, **e**, **g**, **i**) in inflorescence stems. Note the positive signal restricted to the lumen side of xylem when using the *ZeZCP4* promoter in **f** (arrowheads). Magenta color of some pictures represents calcofluor cell wall counterstaining when it was relevant to highlight cell walls. Scale bars: **a**, 20 cm; **b** to **i**: 10 µm.

We first assessed the feasibility of engineering callose deposition into secondary cell walls using the *Arabidopsis thaliana* model. We transformed *Arabidopsis* plants with the *cals3m* construct using three specific secondary cell wall promoters (*pAtIRX8*, *pAtIRX3*, *pZeZCP4* (refs. ^25–28^)) and demonstrated callose synthesis in secondary cell walls via callose immunolocalization (Extended Data Fig. 1 and Supplementary Note 1). Of the three promoters examined, *pAtIRX8* resulted in the broadest callose deposition in xylem and fibres without any clear growth penalties. Consequently, we employed this promoter to drive *cals3m* expression in hybrid aspen (*Populus tremula* × *tremuloides*, clone T89 (refs. ^29,30^), abbreviated ‘WT’ in figures), a leading eudicot model for LB translational research.

**Fig. 1:**
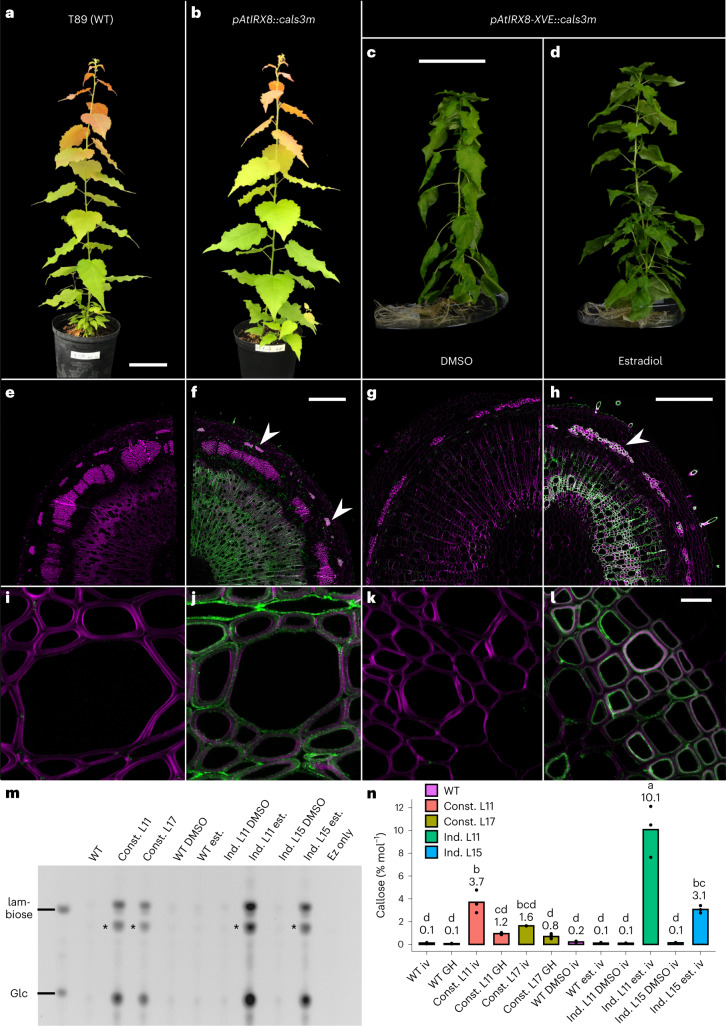
Callose can be effectively and stably integrated in poplar woody biomass. **a**,**b**, Representative pictures of individuals from WT (**a**) and constitutive lines (**b**) after 6-weeks growth on soil in greenhouse conditions (*n* = 5). **c**,**d**, Representative pictures of in vitro-grown inducible line individuals under mock conditions (DMSO, **c**) vs its estradiol counterpart inducing callose synthesis (**d**) 12 weeks after shoot propagation (*n* = 10). **e**–**h**, Callose immunolocalizations on stem cross-sections from individuals presented in **a**–**d**, and their associated close-ups on similar genetic background (**i**–**l**). Callose positive signal is displayed in green (note the signal extending to phloem fibres in transgenic lines in **f** and **h**, arrowheads) and magenta represents calcofluor cell wall counterstaining (**i**–**l**). **m**, PACE callose detection on two independent constitutive (Const.) and inducible lines following estradiol induction (Ind. est.) vs their WT and mock (DMSO) controls. Each migration row represents a pool of five clonal individuals. ‘*’ represents a laminaribiose labelling artefact. **n**, Callose linkage analysis on two independent constitutive (Const.) and inducible lines following estradiol induction (Ind. est.) vs their WT and mock (DMSO) controls. Growth conditions: iv, in vitro; GH, greenhouse. Callose is represented in percentage per mole of total detected linkages. Individual data points represent technical replicates using cell wall extracts obtained by pooling five (GH) or ten (iv) clonal individuals. Statistical analysis was done using one-way ANOVA *(P* = 2.37 × 10^−12^) followed by Tukey’s multiple comparisons test. Significance values for *P* < 0.05 were grouped and are displayed as letter groups above bar plots. Scale bars, 10 cm (**a**,**b**); 5 cm (**c**,**d**); 500 µm (**e**,**f**); 200 µm (**g**,**h**); 10 µm (**i**–**l**). Source data

**Extended Data Fig. 2:**
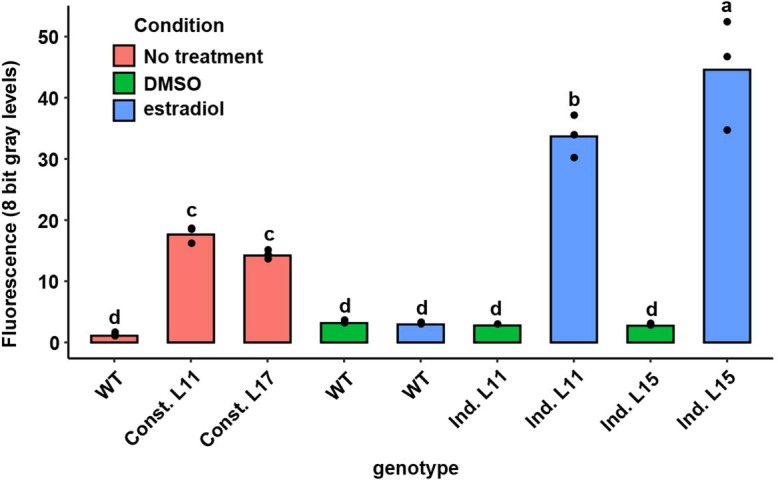
Fluorescence intensity measurements of callose immunolocalizations suggests increased callose deposition in poplar inducible lines when compared to constitutive lines. Average fluorescence measurements of callose immunolocalizations on poplar constitutive (Const.; 6 weeks after soil transfer) and inducible (Ind.; 6 weeks after *in vitro* propagation) lines versus their WT and mock (DMSO) controls. Average fluorescence is shown as grey values out of maximum of 256 (8bits) and has been determined by measuring fluorescence on areas restricted to secondary cell walls using an imageJ macro (full macro script is available in supplementary document 1). DMSO represents the mock treatment for inducible lines and estradiol represents their induced condition. Individual data points represent biological replicates for each line/condition. Statistical analysis was done using one-way analysis of variance (ANOVA, p-value = 2.04e-10) followed by Tukey multiple comparison test. Significance values for P <0.05 were grouped and are displayed as letter groups above the bar plot.

**Extended Data Fig. 3:**
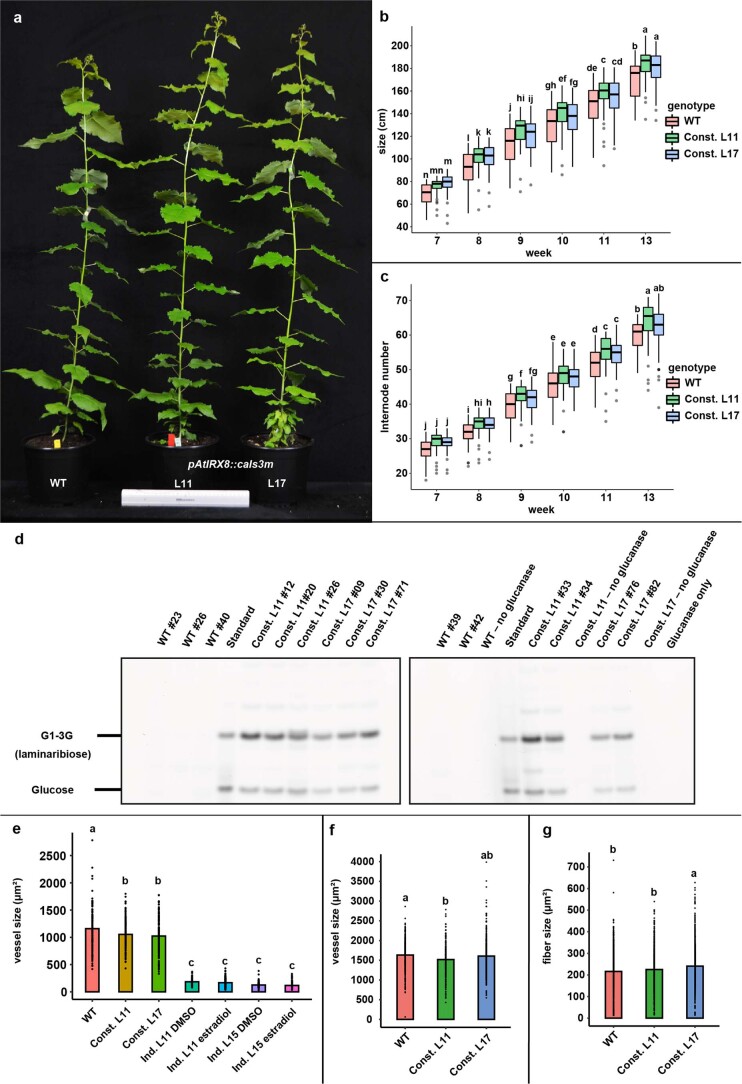
Callose deposition does not result in growth penalty, is stably deposited over time and do not result in major changes in vessels and fibre size. **a** representative tree pictures from a WT and two independent constitutive lines 8 weeks after transfer to soil (Const. L11 and L17, ruler = 30 cm). In the boxplots the black central line indicates the median, the vertical black lines extent to the maximum and minimum values (not considering outliers); grey points represented outliers and the flat ends of the box plot respectively represent the 25th and 75th percentiles. **b** and **c** Average tree size and internode number over time of the same lines, from 7 to 13 weeks after transfer to soil (WT n=67, Const. L11 n=54, Const. L17 n=92. **d** PACE callose detection of two independent constitutive lines (Const. L11 and L17) versus their WT and no glucanase controls, 13 weeks after transfer to soil. Each migration row represents a different individual of a given genetic background (WT, Const. L11 or L17). **e** ImageJ measurement of vessel size in Constitutive (greenhouse grown, 6 weeks after transfer to soil) and Inducible lines (*in vitro* grown, 6 weeks after propagation); 50 vessels were measured for each individual, n = 3 for each genotype/condition. **f** and **g**, vessel and fibre size measurement on greenhouse grown constitutive lines (6 weeks after transfer to soil, n = 5) with LithographX software. A minimum of 248 vessels or 689 fibers were measured for each genotype. Statistical analyses were done using one-way analysis of variance (ANOVA; Extended Data Fig. 4a, p-value = 2e-16; Extended Data Fig. 4b, p-value <2e-16; Extended Data Fig. 4c, p-value = <2e-16) followed by Tukey’s multiple comparison test in **b**, **c** and **e** and with Kruskal Wallis one-way analysis of variance (ANOVA) followed by non-parametric multiple comparison test from nparcomp R package in **f** and **g**. Significance values for P <0.05 were grouped and are displayed as letter groups above bar plots. Source data

Two different constructs were used to transform poplar, *pAtIRX8*::*cals3m* (constitutive lines, abbreviated ‘Const.’ in figures) and its estradiol-inducible version *pAtIRX8-XVE::cals3m* (inducible lines, abbreviated ‘Ind.’ in figures)^31,32^. Representative phenotypes observed for individuals from two independent constitutive lines (greenhouse grown) and inducible lines (in vitro grown) vs their controls are shown in Fig. 1a–d. No visible growth reduction was observed. Similar to *Arabidopsis*, transgenic poplar constitutive and inducible lines showed clear callose deposition throughout xylem vessels and fibres (Fig. 1e–l). Interestingly, callose was also detected in phloem fibres in transgenic lines (Fig. 1f,h, arrowheads). The apparent diminution in primary phloem fibres when comparing the inducible to constitutive lines is probably due to spacing during subsequent tree growth. We then assessed callose accumulation in our poplar lines by comparing relative fluorescence intensities in immunolocalized sections and found signal intensities up to 2.3-fold higher in the inducible lines compared with the constitutive lines (Extended Data Fig. 2). To confirm this observation, we applied polysaccharide analysis through carbohydrate gel electrophoresis (PACE). Callose was specifically hydrolysed from the cell wall extracts with an endo-(1,3)-β-glucanase and the resulting saccharides were separated on a polyacrylamide gel (Fig. 1m). Glucose and laminaribiose were clearly visible only in the genetically engineered lines. Consistent with our immunolocalization results, the bands corresponding to callose hydrolytic products were more intense in the inducible than in the constitutive lines. We also performed glycosidic linkage analysis to quantify callose deposition (Fig. 1n). Here, the proportion of the (1,3)-glucosyl linkages specific to callose is quantified relative to all cell wall glycosidic linkages. The results confirm that the constitutive lines contain callose levels between 0.8 and 3.7%, depending on the line and/or growth conditions considered (greenhouse or in vitro grown), whereas the inducible lines contain (1,3)-glucosyl linkages indicative of callose levels up to 10% of total glycosidic linkages. The stronger callose deposition in the inducible lines probably originates from the design of the estradiol-inducible system, adapted for strong gene expression^31,32^. We then assessed the growth of our constitutive lines in longer greenhouse experiments over the course of 3 months following soil transplantation and did not observe any growth penalty (Extended Data Fig. 3a–c). We also confirm the reproducibility and stability of callose deposition following a longer growth period through PACE analysis (Extended Data Fig. 3d). Finally, we assessed vessel and fibre size in our constitutive lines and inducible lines (Extended Data Fig. 3e–g), which revealed only minor differences solely present in one of the two assessed lines.

To summarize, callose was stably introduced in poplar wood and effectively modulated by taking advantage of the diverse IRX8 promoter strength in the constitutive and inducible contexts. We recognize that further conclusions about the ecophysiological performance of our transformed trees remains to be assessed in field conditions.

### Callose deposition can affect cellulose and lignin contents

**Extended Data Fig. 4:**
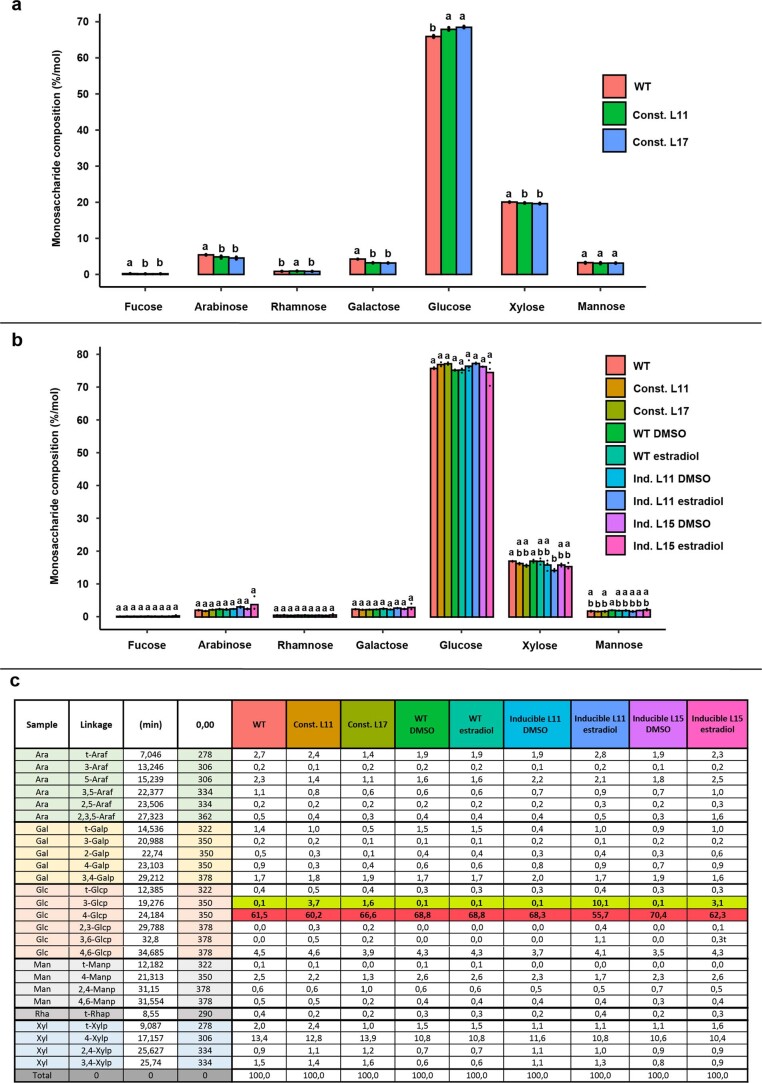
Monosaccharide and linkage analysis of callose enriched cell walls do not show major compositional shift after callose integration. **a** Monosaccharide analysis of poplar cell wall extracts obtained from two greenhouse grown independent constitutive lines (Const.) versus their WT control, harvested 6 weeks after transfer to soil. **b** Monosaccharide analysis of 11 weeks old poplar cell wall extracts obtained from two independent *in vitro* grown constitutive (Const.) and inducible (Ind.) lines versus their WT and mock (DMSO) controls. Additional controls of the WT line with DMSO or estradiol supplemented media are also shown. Individual data points in **a** and **b** represent technical replicates using cell wall extracts obtained by pooling 5 independent individuals of an identical genotype for greenhouse grown lines and 10 individuals for *in vitro* grown lines. **c** Glycosidic linkage analysis of the same poplar material shown in **b**. Results are shown as an average of technical triplicates and are shown in %/mol corrected to the monosaccharide content of the samples. Statistical analyses were done independently for each monosaccharide, either using one-way analysis of variance (ANOVA) followed by Tukey’s multiple comparison test, or with Kruskal Wallis one-way analysis of variance (ANOVA) followed by non-parametric multiple comparison test from nparcomp R package, depending on the Bartlett test result of equality of variances (See Statistical source data for p-value list). Significance values for P <0.05 were grouped and are displayed as letter groups above bar plots. Source data

**Extended Data Fig. 5:**
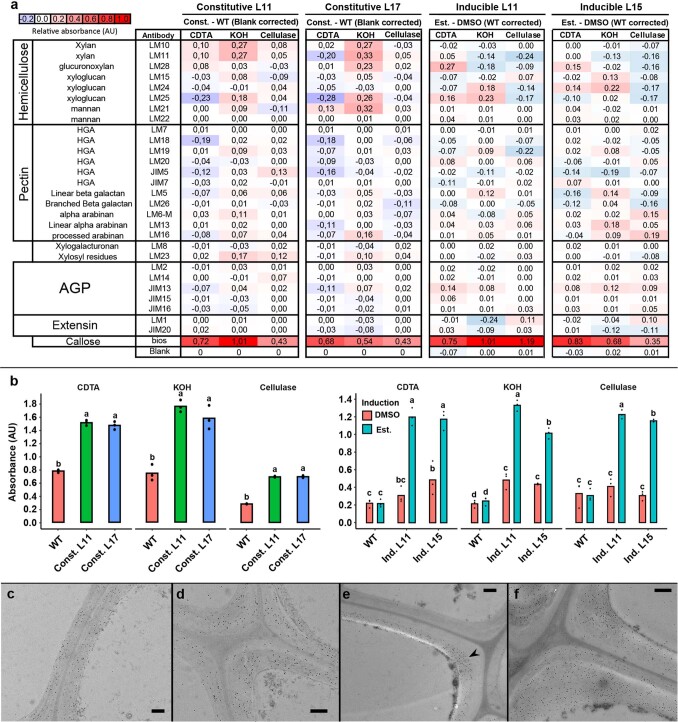
ELISA based Glycome profiling do not show major compositional shift but callose specific ELISA and TEM detection of callose enriched cell walls suggests an integration of callose throughout secondary cell walls. **a** Cell wall glycome profiling table of 11 weeks old poplar cell wall extracts from two independent constitutive (**left**) or inducible (**right**) lines represented as a heat map (see color scale on top left corner) corrected from a WT control, or from mock controls (DMSO) and WT variations observed between estradiol and DMSO growth conditions, respectively. **b** ELISA detection of callose in cell wall extracts of two independent constitutive (Const., **left** panel) or inducible lines following estradiol induction (Ind., **right** panel). The results are represented as absorbance variations bar plots observed between constitutive lines versus their WT controls (**left** panel) or estradiol and DMSO growth conditions (**right** panel). Glycome profiles and ELISA assays were obtained after sequential extractions of the original cell wall extracts (CDTA, KOH, cellulase) and results are shown as average (**a**) or individual data points (**b**) of three technical replicates. Statistical analyses were realized using Kruskal Wallis one-way analysis of variance (ANOVA; Extended Data Fig. 5b, CDTA p-value = 0.06081, KOH p-value = 0.05091, Cellulase p-value =0.06646; Extended Data Fig. 5c, CDTA p-value = 0.01215, KOH p-value = 0.007345, Cellulase p-value = 0.02189) followed by non-parametric multiple comparison test from nparcomp R package. Significance values for P <0.05 were grouped and are displayed as letter groups above bar plots. **c-f** Transmission Electron Microscope pictures of callose immunolocalizations showing callose deposition patterns throughout secondary cell walls in a constitutive line (c, d; Const. Line 11, n=2 biologically independent samples and experiments) and inducible line after estradiol induction (e, f Line 15, n=2 biologically independent samples and experiments). Note the apparition of callose synthesis in panel **e** (arrowhead), corresponding to a secondary cell wall that underwent the transfer of its bearing tree on estradiol inducing media over the course of its biosynthesis. Scale bars: 500 nm.

Primary cell walls are highly reactive to biotic and abiotic stresses through a cell wall integrity system to modulate their composition^33^. On the other hand, it has been suggested that secondary cell walls lack such an intricate system^34^. Therefore, we can hypothesize that the incorporation of an additional polymer to secondary cell walls would not dramatically change its biosynthetic programme. To investigate whether the inclusion of callose could impact the biosynthetic programme and biochemical composition of secondary cell walls, we performed monosaccharide analysis on our engineered wood material. This did not reveal any major changes in composition in either the constitutive or inducible lines (Extended Data Fig. 4a,b). We also assessed cell wall composition through glycosidic linkage analysis (Supplementary Fig. 4c) and found no major differences in the constitutive lines except for changes in (1,3)-Glc linkages indicative of callose (highlighted in yellow) revealed by the specific permethylated alditol acetate 2,4,6-Me_3_-Glc. However, upon activation of callose synthesis in the inducible lines, 1-4 linkages (indicative of cellulose, highlighted in red) appeared significantly reduced. Both callose synthesis and cellulose synthesis require the same pre-cursor (uridine diphosphate glucose); hence it is possible that there may be competition for this resource between the processes^35^. Glycan-specific monoclonal antibodies were then used in enzyme-linked immunosorbent assay (ELISA) to determine the cell wall epitope composition upon callose synthesis in distinct cell wall fractions (pectic, hemicellulosic and cellulosic) following sequential extractions of increasing harshness. No major compositional changes were observed among assessed epitopes (hemicelluloses, pectins and arabinogalactan-proteins (AGPs)), but callose was detected in all cell wall fractions, indicating a strong integration of this ectopic polymer into secondary cell walls (Supplementary Fig. 5a,b and Supplementary Note 2). To visualize callose integration at the ultrastructural level in the engineered wood, callose immunolocalization was performed using transmission electron microscopy (TEM) (Extended Data Fig. 5c–f). In both the constitutive and inducible materials, callose is homogeneously detected throughout the secondary cell walls. Altogether, the ELISA and ultrastructural callose localization experiments suggest a spatial proximity of callose to other secondary cell wall polymers.

**Extended Data Fig. 6:**
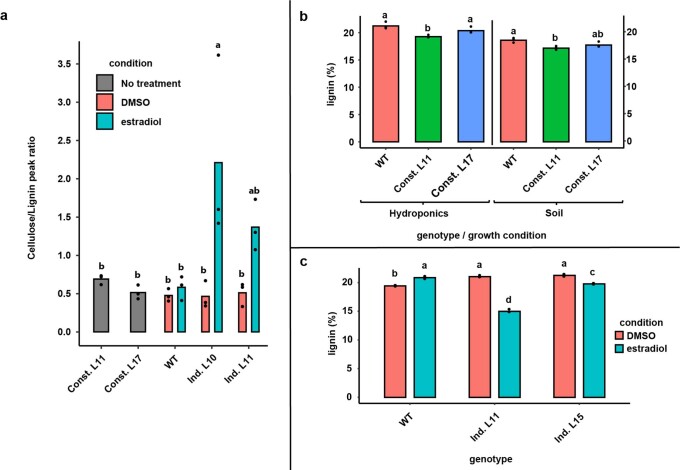
Lignin content is negatively impacted subsequently to callose deposition. **a** Average RAMAN cellulose to lignin ratios between callose accumulating samples in two inducible lines (Ind. L10 and L11, following estradiol induction, 8 weeks of *in vitro* growth) and constitutive lines (Const. L11 and L17, 13 weeks growth after soil transfer), versus non-accumulating ones (Ind. L10 and L11, DMSO mock control) or WT controls (DMSO and estradiol). Each bar plot represents the mean ratio of 3 different clonal individuals for each genotype/condition. For each individual ratio, represented as single data points, a total of 24 points spectra were taken in fibre secondary cell walls in fully differentiated areas at equal distance from the cambium and central pith and averaged before calculating the cellulose to lignin ratios. Prior to averaging, individual spectra were normalized between each other **b**-**c**, average lignin quantification in poplars constitutive and inducible lines by acetyl bromide method. **b** Average lignin quantification of two constitutive lines (Const.) versus WT poplars grown in two different conditions (hydroponics, 8 weeks after rockwool transfer; soil grown, 13 weeks after soil transfer). **c** Average lignin quantification of two inducible lines (Ind.) versus their mock controls (DMSO) and estradiol and DMSO treated WT poplars grown in hydroponics conditions, 8 weeks after rockwool transfer. Individual data points represent technical replicates using stem samples obtained by pooling 5 independent individuals of identical genotypes. Statistical analyses were done using one-way analysis of variance (ANOVA; Extended Data Fig. 6a, p-value = 2.26e-3;) followed by Tukey’s multiple comparison test in **a** and with Kruskal Wallis one-way analysis of variance (ANOVA; Extended Data Fig. 6b, Hydroponics p-value = 0.05091, Soil p-value = 0.07939, Extended Data Fig. 6c, p-value = 7.787 e-3) followed by non-parametric multiple comparison test from nparcomp R package in **b** and **c**. Significance values for P <0.05 were grouped and are displayed as letter groups above bar plots.

Finally, we assessed lignin polymers in callose engineered wood using RAMAN spectroscopy (Extended Data Fig. 6a). Surprisingly, the cellulose to lignin peak ratios were reduced in two independent inducible lines (Ind. L10 and Ind. L11) and showed a slight decrease in one of our constitutive lines (Const. L11). To confirm this, we performed a quantitative lignin analysis (Extended Data Fig. 6b,c). A small but consistent decrease in lignin (−5 to −9%) was observed in the constitutive lines both in hydroponics- and greenhouse-grown plants and a strong lignin decrease in the inducible line 11 (−29%). This suggests a negative correlation between lignin and callose accumulated in the secondary cell walls.

### Callose increases cellulose–lignin distance and cell wall hydration

**Extended Data Fig. 7:**
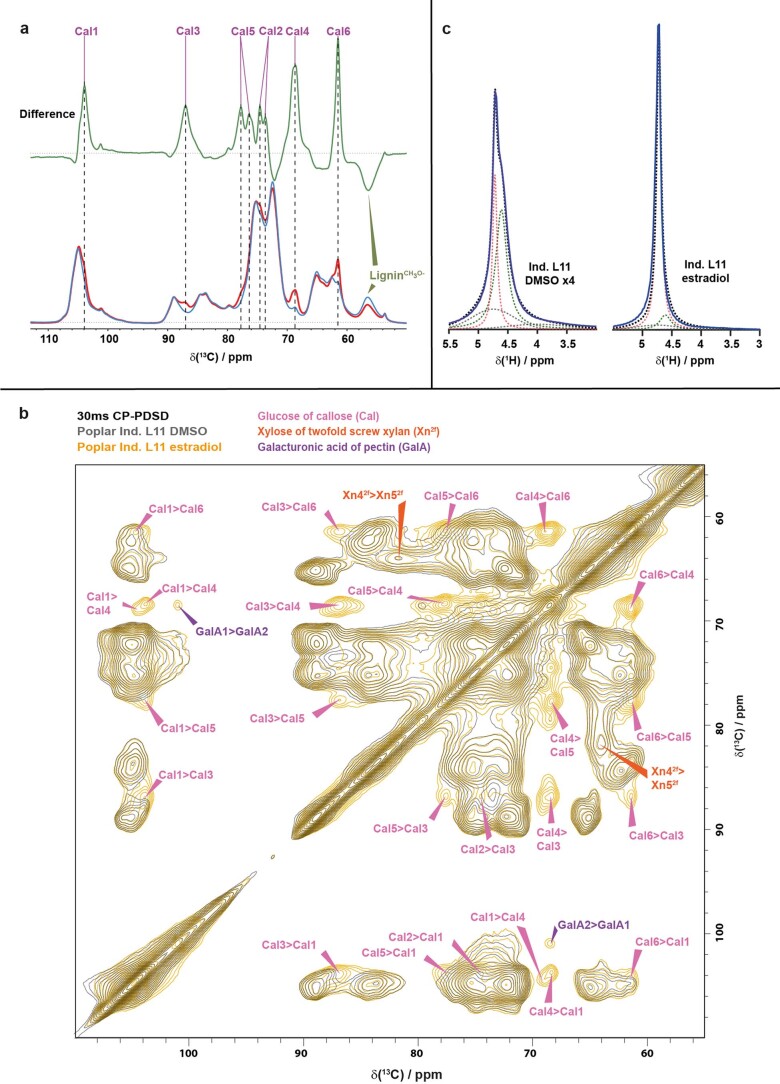
ssNMR detects for the first time the presence of callose *in muro* and reveals an increase of free water in callose enriched woody biomass. **a** ^13^C NMR assignment of Callose from 1D ^13^C CP MAS spectra: the callose carbon peaks can be revealed by taking the difference between a callose accumulating sample (inducible L11, estradiol induced during 14 weeks) versus a non-accumulating one (inducible L11, DMSO mock control). The individual 1D ^13^C CP MAS spectra of the estradiol (red) and DMSO (blue) conditions are shown below the difference spectrum (green). The lignin peak, which shows reduced lignin in the callose accumulating sample, is also indicated. **b** Overlay of 30 ms mixing time ^13^C PDSD NMR spectra of a callose accumulating sample (inducible L11, estradiol, yellow) versus a non-accumulating one (inducible L11, DMSO, black) is shown. All callose peaks for the two callose environments are visible and highlighted (see for example Cal1 at ~ 103, 68 ppm) and their shifts are summarized in Supplementary Table 1. Xylan (Xn, orange) and Galacturonic acid of pectin (GalA, purple) are also highlighted. **c** ^1^H spectra of poplar inducible Line 11 shows a dramatic increase amount of ‘free’ water when comparing the induced (estradiol, **right**) versus uninduced (DMSO, **left**) condition. Spectra were acquired at 500 MHz, spinning at 12.5 kHz, using an echo with the echo spacing of one rotor period (80 µs) to reduce background and polysaccharide signals. The bound and ‘free’ water are simulated using dmfit^90^ by Lorentzians and the background by Gaussians. Red Lorentzian is the ‘free’ water; green Lorentzian is the bound water; whilst the grey Gaussian is the residual background signal from polysaccharides and probe.

The homogeneous integration of callose in secondary walls raises the question of its interaction with other cell wall polymers and equally important, its potential to disrupt existing interactions between the core cell wall polymers. To assess this, we performed solid-state nuclear magnetic resonance (ssNMR) experiments on never-dried wood samples, a technique that has recently proven to give invaluable insight on how polymers tightly interact in cell walls^36–38^. Transgenic inducible poplars were grown in vitro in a ^13^CO_2_ atmosphere and ^13^C glucose supplemented medium^36^. Callose synthesis was induced with estradiol supplemented media and compared to their corresponding mock controls (dimethylsulfoxide (DMSO) supplemented media). One-dimensional (1D) cross polarization (CP) magic-angle spinning (MAS) NMR, which emphasizes the more rigid cell wall elements, clearly shows the extra peaks corresponding to callose on a difference spectrum (green, Extended Data Fig. 7a), obtained when subtracting the induced (red) vs control spectrum (blue). Callose carbons were assigned in the spectrum by comparison with shifts previously determined by solid-state ^13^C CP MAS on isolated (1,3)-β-glucans^16,39,40^. Interestingly, callose carbons 2 and 5 appear to display two distinct chemical shifts in the 1D NMR difference spectrum. This spectrum also confirmed a decrease in lignin in the induced sample containing callose (Extended Data Fig. 7a, green arrowheads). To better resolve the callose signal, a short mixing time (30 ms) 2D ^13^C CP proton driven spin diffusion (PDSD) experiment was performed. In this experiment, magnetization is transferred to nearby ^13^C nuclei by dipole coupling, with cross peaks being observed between spatially close carbons, such as those within the same monosaccharide residue of a polysaccharide chain. Extended Data Fig. 7b shows an overlay of the induced (estradiol, yellow) and mock control (DMSO, black) 2D 30 ms CP PDSD spectra in which the callose signals are observed and where callose, galacturonic acid and twofold xylan cross peaks are highlighted. Careful inspection of the data confirms that there are two distinct sets of callose shifts in a 1:1 ratio in the estradiol-induced sample, with callose carbons 1 and 4 (see for example, Cal 1 near 104 ppm, Extended Data Fig. 7b) as well as carbons 2 and 5 being well resolved (Supplementary Table 1).

**Fig. 2:**
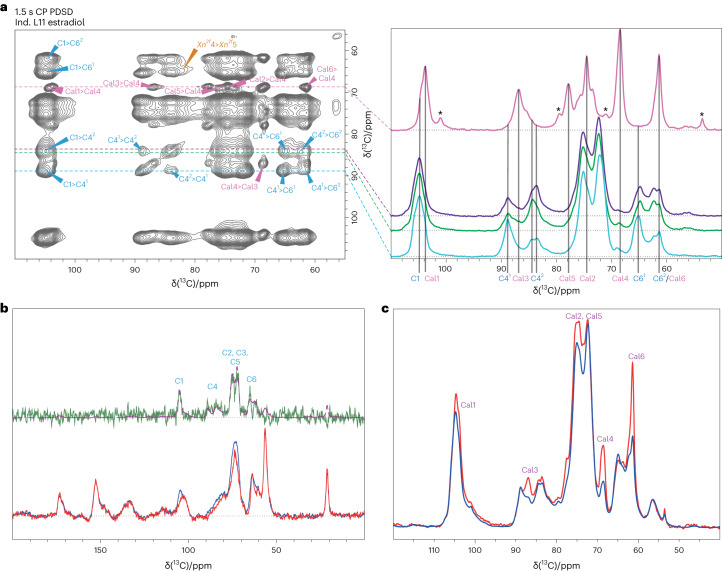
ssNMR reveals that callose is close to water, is not close to other secondary cell wall polymers and increases the lignin to cellulose distance. **a**, Left: 1.5 s mixing time ^13^C CP PDSD NMR spectrum of an induced poplar sample (inducible L11 following estradiol induction for 14 weeks). Right: 1D slices taken from the 1.5 s mixing time PDSD spectrum. A slice taken through the callose Cal4a shift at 68.5 ppm (pink) is compared with slices taken at different cellulose environments. The cellulose slices are C4^1^ at 88.7 ppm (light blue), C4^2^ at 84.3 ppm (green) and a further C4^2^ at 83.7 ppm (purple). The additional peaks, marked with ‘*’, seen in the callose slice come from galacturonic acid whose C2 carbon has a shift of ~68.8 ppm very similar to callose C4. The barely resolvable peaks at ~73.0 and 75.7 ppm in this slice come from the second callose environment. The slices show that the callose is further than 10 Å from both cellulose and hemicellulose. **b**, Slices from the 1.5 s PDSD through the lignin peak at 56 ppm of the inducible L11 following estradiol induction (red) vs its DMSO mock control (blue) and the difference (green). The difference reveals intensity at all cellulose shifts as shown when overlaying a CP MAS spectrum over the difference spectrum (mauve) indicating that the lignin is closer to cellulose in the DMSO mock control sample. **c**, A comparison of the water-edited ^13^C CP NMR spectrum of induced (L11) poplar with a diffusion time of 4 ms (red) and the normal CP spectrum (blue) normalized to the cellulose C4 at 89 ppm. The increased relative intensity of the callose peaks shows that it is close to water.

We then probed the longer distance environment of callose with a 1.5 s mixing time CP PDSD experiment (Fig. 2a), which probes intermolecular distances out to ~0.7–1.0 nm. Surprisingly, no interactions between callose and other secondary cell wall polymers (cellulose, xylan and lignin) were detected, even though the two cellulose domains clearly show cross peaks with one another. The cellulose domains 1 and 2 approximate to interior and surface residues^37,41^ and represent 39% and 61% of the cellulose in our dataset. The right-hand side of Fig. 2a shows slices taken at the callose Cal4 shift at 68.5 ppm (pink line), at the cellulose domain 1 C4^1^ shift (88.7 ppm, blue) and at domain 2 C4^2^ at 84.3 ppm (green) and 83.7 ppm (purple). The C4^2^ cellulose surface sites are clearly visible in the interior C4^1^ slice at 88.7 ppm and vice versa, whereas there does not seem to be any interaction visible in the callose Cal4 slice (peaks at ~101, 79, 53.7 ppm and so on, originate from galacturonic acid residues in pectins, whose C2 carbon has a chemical shift at 68.8 ppm very close to that of callose Cal4, hence its signal also appears). Interestingly, when comparing the lignin slices at 56 ppm (normalized to the lignin signal), we noted that lignin and cellulose are spatially further apart in the induced than in the uninduced sample, since cellulose peaks appear in the difference spectrum (Fig. 2b). This implies that not only is there an impact of callose on lignin content, but its presence also effects the lignin–cellulose distance within the cell wall matrix.

We also assessed the relative proximity of callose to water using a water-edited experiment^42,43^ in which a ^1^H *T*_2_ filter is used to remove any direct contribution to the CP spectrum from the polysaccharide protons. Only polarization from mobile protons, that is, from water protons, is left after the filter. A variable delay is then used to allow the ^1^H magnetization to diffuse from the water to the polysaccharide protons; this magnetization is then transferred to the nearest ^13^C by a standard CP experiment, permitting assessment of the relative proximity of water to nearby polysaccharides. Figure 2c shows a comparison between the water-edited ^13^C CP MAS NMR spectrum of the inducible L11 following estradiol induction (red) and the normal CP spectrum (blue), with a diffusion time of 4 ms. The spectra are normalized to the cellulose C4 at 89 ppm. The callose peaks appear much more intense in the water-edited spectrum, showing that it is closer to water than cellulose. In addition, a ^1^H echo spectrum of the two samples revealed that there is ~4.5 times more water in the estradiol-induced sample than in the DMSO mock control (Extended Data Fig. 7c). Interestingly, the water detected in the induced sample is almost all in one environment, whereas the control has two water environments: one similar to the induced condition and the other with a slightly different shift, that is, broader and has a significantly shorter *T*_2_, indicating more tightly bound/confined water (Extended Data Fig. 7c and Supplementary Table 2).

### Callose increases cell wall porosity and enzymatic hydrolysis

**Fig. 3:**
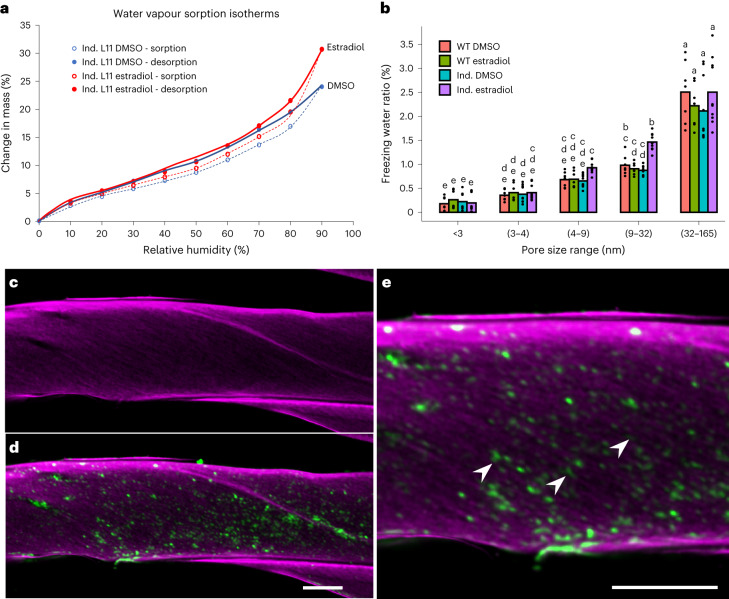
Callose synthesis provokes an increase in cell wall hygroscopicity and porosity through its deposition in cell wall spaces. **a**, Water vapour isotherms and Park model representation of inducible L11 following estradiol vs a mock (DMSO) control after 14 weeks of growth. **b**, DSC thermoporosimetry histogram showing the distribution of pore diameter in association with the ratio of freezing water (in percentage) using the inducible (Ind.) and WT backgrounds in different conditions (DMSO mock and estradiol-inducing condition) following 14 weeks of growth. The higher the percentage is, the higher a range of pore diameters is represented in the considered lignocellulosic biomass. Individual data points represent biological replicates in DMSO (WT DMSO *n* = 7, Ind. DMSO *n* = 10) or estradiol conditions (WT estradiol *n* = 7, Ind. estradiol *n* = 10). For inducible lines, results of two independent lines were pooled in each bar. Statistical analysis was done using one-way ANOVA (*P* = 2 × 10^−16^) followed by Tukey’s multiple comparisons test. Significance values for *P* < 0.05 were grouped and are displayed as letter groups above bar plots. **c**, Representative cellulose deposition in a longitudinal view of a poplar fibre cell revealed by Direct Red staining (magenta) from inducible lines following estradiol-induced callose synthesis during 14 weeks. **d**, Same as **c** but merged with callose immunolocalization to reveal the integration pattern of the ectopic polymer. **e**, Close-up of **d**. Arrowheads indicate spots of callose deposition in cellulose gaps. Two independent lines (*n* = 3) were used for this experiment. Scale bars, 5 µm.

Given the overall enrichment of mobile water following callose deposition, we wanted to assess further water behaviour in our engineered woody biomass. To this end, we performed dynamic vapour sorption (DVS) analysis. DVS is a gravimetric sorption technique that measures how quickly and how much of a solvent (in this case water) is absorbed by a sample (Supplementary Note 3 and Supplementary Fig. 1a,b). Here, the wood samples were subjected to gradual increase or decrease in relative humidity (RH) during consecutive sorption and desorption cycles and their weight change measured at mass equilibrium. The DVS analysis shows a specific increase in mass gain in the 70–90% RH zone for the callose-enriched wood. Specifically, a 30% mass gain was observed for the callose-enriched sample (estradiol induced), whereas the mass gain for the mock control (DMSO) amounted to 24% at the 90% RH sorption point (Fig. 3a). In DVS, the 70–100% RH zone reflects the clustering of water molecules at equilibrium state and is related to the capillary behaviour of a material in relation to its mesoporosity (2–50 nm pore range^44^). Together, our results strongly suggest that callose improves water absorption and the mesoporosity of lignocellulosic biomass (Supplementary Note 3 and Supplementary Fig. 1c). We then specifically assessed changes in wood porosity following callose deposition using differential scanning calorimetry (DSC) thermoporosimetry^45^. The technique relies on water exhibiting different thermodynamic behaviour when in contact with different surfaces and on the fact that water crystals located in porous structures require more energy to melt^46^. This translates to water melting enthalpies occurring at lower temperature than the usual 0 °C. When approximated to perfect cylinders, the size of the pores can be extracted from their melting point with DSC analysis^45^. Interestingly, when we triggered callose deposition via estradiol treatment, the proportion of pores in the 4–32 nm range was significantly increased (Fig. 3b). This confirms that ectopic callose synthesis in woody biomass can effectively alter its mesoporosity.

**Extended Data Fig. 8:**
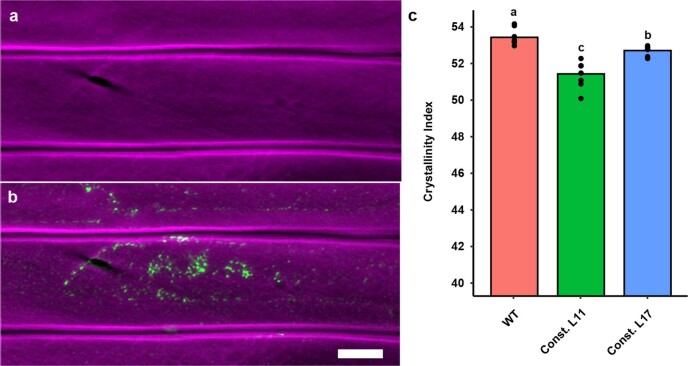
Callose is integrated in cell wall pores and provoke a decrease in biomass crystallinity in constitutive lines. **a** Representative cellulose deposition in a longitudinal view of a fibre cell revealed by Direct Red staining (magenta) on constitutive lines, 13 weeks after transfer to soil. **b** Same as **a** but merged with callose immunolocalization to reveal the integration pattern of the ectopic polymer. Two independent lines (n = 3) were used for this experiment. Scale bar: 5 µm **c** Wood crystallinity assessment by X-Ray diffraction represented as Crystallinity indexes of two independent constitutive (Const.) lines versus their WT controls. Greenhouse grown wood samples were harvested 3 months after transfer to soil. Individual data points represent biological replicates of 8 individuals for each genotype. Statistical analysis was done using Kruskal Wallis one-way analysis of variance (ANOVA, p-value = 3.92e-05) followed by non-parametric multiple comparison test from nparcomp R package. Significance values for P <0.05 were grouped and are displayed as letter groups above the bar plot.

The effect of callose synthesis on cell wall capillarity and porosity prompted us to reassess callose localization. We performed callose immunolocalizations on our inducible (Fig. 3c–e) and constitutive lines (Extended Data Fig. 8a,b), in sections taken longitudinally to xylem vessels and fibres. Interestingly, callose is in spaces between cellulose microfibril aggregates in a discrete punctuated pattern. This suggests that the observed effect on wall porosity could be mediated by a physical distancing of cellulose microfibril aggregates in their longitudinal axis through callose deposition. From this observation, we wanted to assess callose capacity to impact wood crystallinity. To this end, we performed X-ray diffraction experiments in callose-enriched wood using greenhouse-grown constitutive lines as wood samples. The results show a slight but consistent decrease in crystallinity index (Extended Data Fig. 8c), suggesting that callose deposition in secondary cell walls can decrease overall lignocellulose crystallinity.

**Extended Data Fig. 9:**
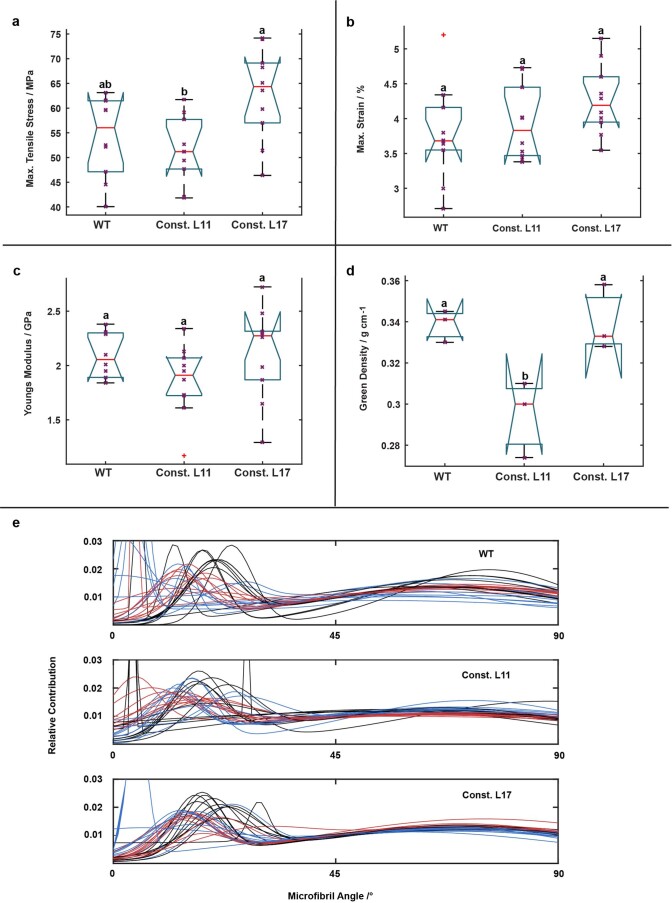
Callose deposition does not alter density, microfibril angle and tensile properties. **a-d** Summary of wood mechanical properties for two constitutive lines (Const.) compared to WT, grown 13 weeks after transfer to soil. **a** Maximum tensile stress, **b** maximum strain, **c** Young’s Modulus, **d** green density. In the boxplots the red central line indicates the median, the black whiskers extent to the maximum and minimum value respectively (not considering outliers); individual purple data points represent the average of all measurements performed within a single tree individual, and outliers are represented by the red “+” symbol; the flat ends of the blue box plot represent the 25th and 75th percentiles respectively; the blue notch extremes indicate the q2 – 1.57(q3 – q1)/sqrt(n) and q2 + 1.57(q3 – q1)/sqrt(n), with q2 being the median, q1 and q3 the 25th and 75th percentiles, respectively, and n the number of observations without any NaN values. Statistical analysis, using a one-way analysis of variance (ANOVA, Extended Data Fig. 9a, p-value = 1.05e-02; Extended Data Fig. 9b, p-value = 0.1769; Extended Data Fig. 9c, p-value = 0.1729; Extended Data Fig. 9d, p-value = 1.58 e-02) and subsequent comparison tests, were performed on MATLAB. Significance values for P <0.05 were grouped and are displayed as letter groups above boxplots. **e** The microfibril angle distribution in the cell wall S2 layer was determined using X-ray diffraction for a specimen in the periphery of the stem (black lines), one specimen from the center of the stem (red lines) and for one specimen from a position in-between (blue lines) for WT (top), Const. L11 (middle) and Const. L17 (bottom). The microfibril angle distribution did not substantially differ between WT, Const. L11, and Const. L17.

Changes in cell wall porosity and cell wall component interactions may have an impact on wood mechanics. To assess this, we performed tensile tests on wet wood specimens (previously dried upon sampling) from the constitutive lines vs a wild-type reference (Extended Data Fig. 9a–c). Interestingly, no major changes in tensile stiffness and strength could be observed despite the structural and compositional changes to the cell wall caused by callose deposition. Statistical analysis of biological replicates revealed only one significant difference in mechanical properties, namely between the maximum tensile stress of constitutive line 11 and line 17, without any greater deviation from WT. The entire removal of water can result in the formation of a substantial amount of micro- and nanocracks in the wood structure leading to irreversible changes in fibril nanostructure^43^ and having influence on wet mechanical properties. However, our samples were previously dried at ambient indoor climate conditions, resulting in an approximate moisture content of 8–10% (vs 0% for oven dried) before re-wetting for cutting and tensile testing. Thus, we propose that this milder drying process has only little impact on the wood structure and wet mechanical properties and is unlikely to superimpose potential mechanical differences. To rule out superimpositions by further potential structural alterations, we performed green density measurements (Extended Data Fig. 9d) and measured microfibril angles (MFA, Extended Data Fig. 9e) in the S2 layers of the cell walls by X-ray diffraction, since density and MFA are known to strongly affect the tensile properties of wood tissues^47^. MFA distribution was not altered by callose deposition, while density showed significant differences between WT and line 11, and between lines 17 and 11. The lower density of line 11 compared with WT and line 17 is in accordance with the slightly, but not significantly, lower maximum tensile stress measured for this line. Since there is no consistent alteration of structural and mechanical properties of the two constitutive lines compared to the wild type and even the significant differences are in a range of common variability between biological replicates, we conclude that deposition of callose in the cell walls does not result in a tensile strength or stiffness penalty. In wood samples, the uptake of water in the cell walls already results in an increased distance between cellulose fibrils and weakening of intermolecular interactions. Hence, the further alteration in water adsorption and distribution following callose synthesis may not have a measurable effect on the stress transfer mechanism between cellulose fibrils and matrix polymers.

**Fig. 4:**
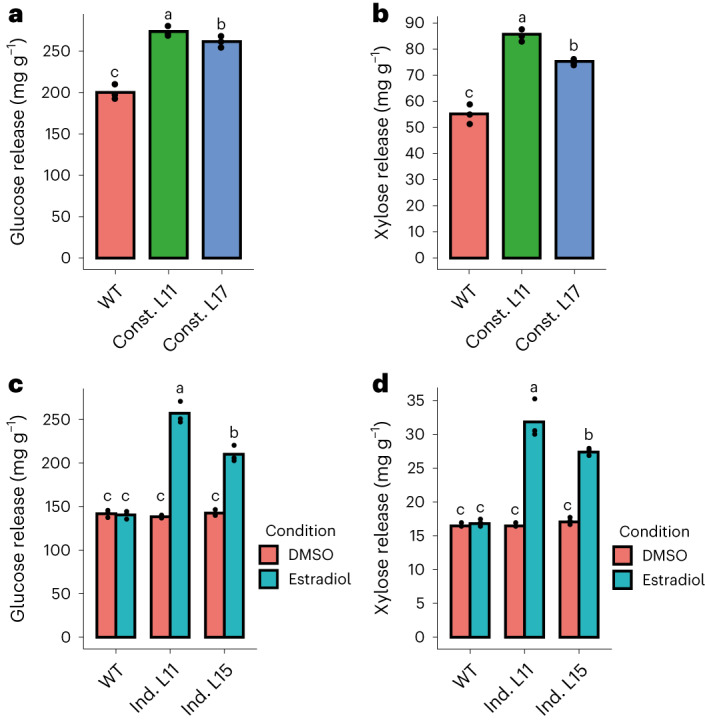
Callose-enriched biomass shows improved saccharification yields positively correlated with callose amounts deposited. **a**,**b**, Average glucose (**a**) and xylose (**b**) release following a 72 h saccharification of cell wall extracts from 2 independent constitutive lines (Const.) vs a WT control harvested 6 weeks after transfer to soil. **c**,**d**, Average glucose (**c**) and xylose (**d**) release following a 72 h saccharification of cell wall extracts from 2 independent inducible lines (Ind.) grown on estradiol-inducing media for 12 weeks vs their WT and mock controls (DMSO). Individual data points represent technical replicates using cell wall extracts obtained by pooling five independent individuals of an identical genotype for greenhouse-grown lines (**a**,**b**) and ten individuals for in vitro-grown lines (**c**,**d**). Statistical analysis was done using one-way ANOVA (*P* = 4.55 × 10^−9^ (**d**)) followed by Tukey’s multiple comparisons test for xylose release in inducible lines, and with Kruskal–Wallis one-way ANOVA (*P* = 0.02732 (**a**), *P* = 0.02732 (**b**), *P* = 0.02375 (**c**)) followed by non-parametric multiple comparisons test from the nparcomp R package for the rest of analysed conditions. Significance values for *P* < 0.05 were grouped and are displayed as letter groups above bar plots.

**Extended Data Fig. 10:**
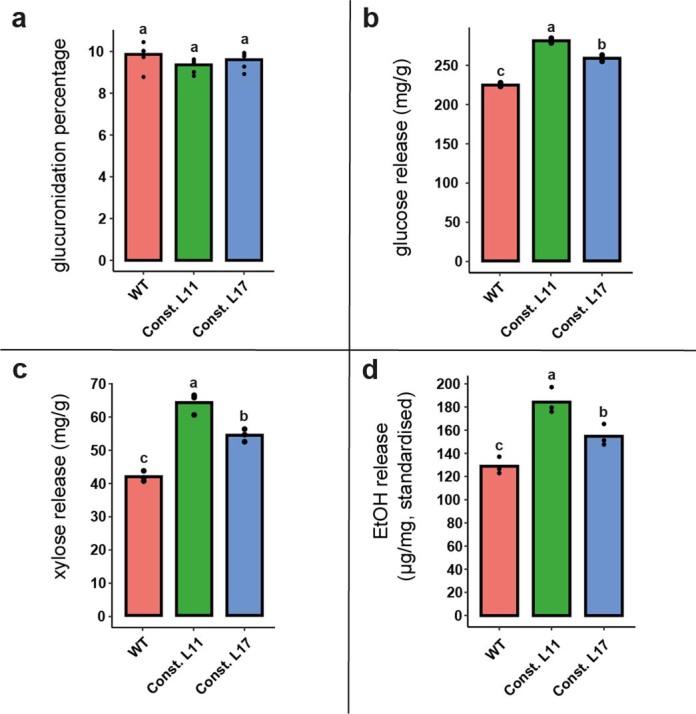
Callose deposition does not alter xylan glucuronidation and leads to improved saccharification and ethanol production. **a** Histogram showing xylan glucuronidation degrees quantified using ImageJ software from PACE band intensity measurement. Individual data points represent biological replicates of 5 individuals for each genotype. **b-c** Average glucose and xylose release following a 72h saccharification. **d**, relative quantity of ethanol release in mg/g of biomass, standardized with ethanol release from the enzymatic Ctec mix only and no Ctec condition for each genotype. Cell wall extracts of two independent constitutive lines (Const.) versus WT controls, harvested 4 months after the tree transfer to soil, were used in those experiments. Individual data points represent technical replicates of three technical replicates using cell wall extracts obtained by pooling 5 independent individuals of identical genotypes (constitutive lines versus WT). Statistical analyses were done using Kruskal Wallis one-way analysis of variance (ANOVA) followed by non-parametric multiple comparison test from nparcomp R package (Extended Data Fig. 10a, p-value = 0.208; Extended Data Fig. 10b, p-value = 0.02732; Extended Data Fig. 10c, p-value = 0.02732; Extended Data Fig. 10d, p-value = 0.02732). Significance values for P <0.05 were grouped and are displayed as letter groups above bar plots.

As callose deposition is accompanied by increased LB porosity, capillarity and crystallinity, we hypothesize that this would lead to an increased accessibility to cell wall degrading enzymes in our engineered wood, which may result in a decrease in its recalcitrance to enzymatic hydrolysis. To assess this, we measured enzymatic saccharification efficiency on our engineered poplar biomass. In the constitutive lines, sugar release was increased by up to 37% and 55% for glucose and xylose, respectively (Fig. 4a,b). For the inducible lines, increases in saccharification efficiency were even more pronounced, with monosaccharide release being up to 81% higher for glucose and 94% higher for xylose (Fig. 4c,d). This suggests that saccharification yield improvement is positively correlated with the amount of callose integrated in the cell wall. Interestingly, xylose release (predominantly related to xylan hydrolysis in hybrid aspen wood samples) is also significantly improved in all callose accumulating lines. As lower xylan glucuronidation levels were previously reported to improve xylan accessibility to enzymatic hydrolysis^48,49^, we probed for any potential effect of callose synthesis on xylan glucuronidation levels in our constitutive lines. No significant effect was observed on the overall glucuronidation levels (Extended Data Fig. 10a), suggesting that the observed effect is specific to changes in cell wall molecular architecture caused by callose deposition.

Finally, to assess the suitability of our approach to benefit specific LB transformation chains, we used biomass from the constitutive lines as feedstock for bioethanol production. We first confirmed that the saccharification phenotype is maintained for wood sourced from a longer growth period (13 weeks, greenhouse conditions). For this biomass, sugar release was increased by up to 25% and 53% for glucose and xylose, respectively (Extended Data Fig. 10b,c). When using the same engineered feedstock for bacterial simultaneous saccharification and fermentation (SSF)^49,50^, we observed an increase in bioethanol production of up to 42% (Extended Data Fig. 10d). Together, these results demonstrate that integration of callose into the secondary cell wall leads to large increases in both saccharification and bioethanol production. In constitutive and inducible materials, the gains in sugar release and consequently, ethanol, are clearly greater than any gains that would be directly attributable to the absolute fermentable sugar introduction to the wall through callose synthesis, accounting for a maximum of 1–10% of total sugars (constitutive vs inducible lines, respectively). This strongly suggests that the observed effect on recalcitrance can be attributed to the impact callose has on secondary cell wall porosity, crystallinity, hydration capacity and potentially lignin content.

## Discussion

### A model for callose integration in secondary cell walls

**Fig. 5:**
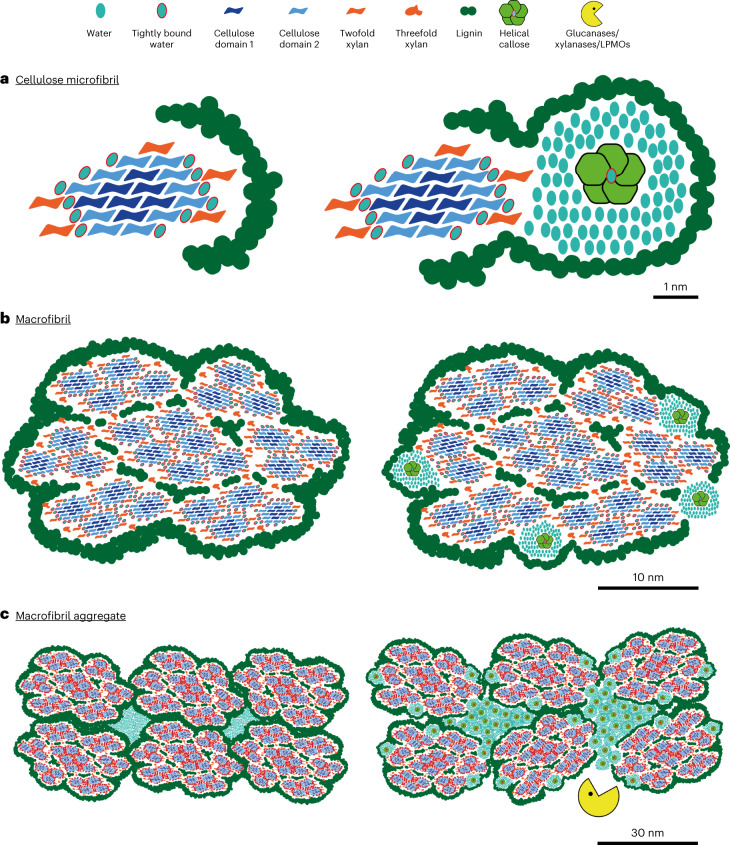
Model of action for callose integration in lignocellulosic biomass. Callose acts as a cell wall spacer subsequently impacting biomass porosity and enzymatic access through an increase in hygroscopicity. **a**, Model of a poplar cellulose microfibril without (left) and with callose deposition (right). Callose is represented as a cross section of a helical structure with central bound water surrounded by mobile water, which partially increases the distance of lignin from cellulose. Bound and mobile water around callose explain the absence of short-range interactions between callose and other secondary cell wall polymers. **b**, Model of a poplar macrofibril without (left) and with callose deposition (right). Callose is inserted in between coalesced microfibril groups. The deposition of callose negatively impacts the subsequent polymerization of lignin surrounding the macrofibril. For representation convenience, we decided to represent the higher end of the poplar macrofibril size (30 nm) according to ref. ^53^. **c**, Model of poplar macrofibril assembly without (left) and with callose deposition (right). Callose self-aggregates in between macrofibrils, which explains the observed increase in secondary cell wall porosity. The range of pore size affected is 4–30 nm, which is in the size range of hydrolytic enzymes. As such, callose is believed to act as a hydrophilic spacer of secondary cell wall polymer, further promoting access to hydrolytic enzymes for subsequent saccharification. LPMOs, lytic polysaccharide monooxygenases.

We have demonstrated the possibility of integrating callose into eudicot woody biomass. More importantly, the multidisciplinary analysis implemented in this study allowed us to assess the integration of callose into secondary cell walls at a nano scale. From the results obtained, we propose a model of cell wall assembly, from microfibrils to macrofibril assembly, with and without callose (Fig. 5), which summarizes the impact of callose deposition on cell wall local structure and accessibility to enzymatic hydrolysis.

At the elementary fibril scale (Fig. 5a, left), the native tree cellulose microfibril is displayed as an 18-glucan-chain assembly in a 234432 configuration, an increasingly accepted model in secondary cell walls^43,51^. Our monosaccharide analysis and ssNMR data allowed us to build a stoichiometric model of polysaccharide distribution between xylan, cellulose and cellulose domains. Based on the use of the *AtIRX8* promoter, related to xylan synthesis, callose deposition most probably precedes lignin polymerization^25^. Thus, we hypothesize that this early deposition is the main driver for the average distance increase between lignin and cellulose shown by the ssNMR data (Fig. 5a, right). Callose is represented as a right-handed sixfold helical hydrated conformation, with a 1.56-nm helix diameter as previously determined^13^. Our ssNMR experiments represent the first *in muro* detection of callose polymers and reveal that callose is present in two different conformations or NMR environments (Supplementary Table 1), and these data can suggest its potential structural conformation *in muro*. These two environments, most obvious through specific variations in the C1, C2 and C5 chemical shifts, are almost identical to those observed for hydrated (peak A) and hydrothermal annealed (peak B) curdlan (an extracellular bacterial (1,3)-β-glucan)^16,14^ (see Supplementary Table 1). The hydrothermal annealed curdlan is known to have a triple helical chain structure and a similar helical chain structure is very likely for the hydrated form^16^. Thus, we propose that callose in our engineered wood is also in a highly hydrated triple helical conformation. Given the high proportion of these hydrated allomorphs in our data, along with the close proximity of callose to free water, we suggest that the high hydrophilicity of callose attracts enough surrounding water that the distance is too large for the NMR signal to be transferred from callose to other wall polymers (Fig. 5a, right).

In secondary cell walls, cellulose microfibrils are assembled together with xylan and lignin into microfibril aggregates or macrofibrils^52–54^, and the poplar native macrofibril is here represented at scale with our previous observations^53^ (Fig. 5b, left). We suggest that cellulose microfibrils coalesce together from the observed coordination of cellulose synthase complexes at the plasma membrane^55–57^. Threefold xylan (~20% of total xylan in our ssNMR data) is thought to interact directly with lignin^43,51,54^ and is thus inserted between coalesced microfibril groups and surrounding the macrofibril assembly (Fig. 5b, left). When incorporating callose in the model at the macrofibril scale (Fig. 5b, right), we propose that this mostly occurs around the macrofibril assembly before lignin synthesis, perhaps leading to the observed decrease in lignin and the average increase in lignin–cellulose distance. We also observed a slight decrease in cellulose content when a significant amount of callose is produced. Since callose and cellulose synthases share the same uridine diphosphate glucose substrate^35^, it is possible that the observed cellulose decrease in callose-enriched walls is the result of competition between the concomitant cellulose and callose synthase activities for a common substrate pool. In our macrofibril model, these data are reflected by fewer lignin polymers in the vicinity of callose units and fewer cellulose microfibrils (Fig. 5b, right).

Finally, we show the putative conformation of macrofibril aggregates (Fig. 5c, left). At this scale, macrofibrils interact, leaving small gaps in the LB representing its mesopores, where the bulk of free water within cell walls is thought to be present (excluding intraluminal free water in vessels and fibres). We demonstrate that callose deposition increases the proportion of pores in the 4–32 nm range and our ssNMR analysis indicates distances >1 nm between callose and other secondary cell wall polymers. From this observation, we suggest that the bulk of synthesized callose is deposited between cell wall macrofibrils and self-aggregates in the spaces among them (Fig. 5c, right), in accordance with our callose detection in longitudinal cell walls (Fig. 3d,e and Extended Data Fig. 8a,b). The callose layer around the endosperm of muskmelon seeds has been previously reported to show properties of a semi-permeable layer^58^. If callose is mostly deposited within cell wall pores, its semi-permeable properties may impact the diffusion of monolignols and/or laccases and peroxidases necessary for subsequent lignin polymerization while keeping water imbibition properties. Therefore, we displayed less lignin polymers in the vicinity of callose aggregates between the cell wall macrofibrils in our model (Fig. 5c, right). In addition to its semi-permeable properties, callose was previously reported to be easily hydrated, which results in strong swelling^59^. This fits with our ssNMR data which also highlights the highly hydrated nature of callose *in muro*^16^. Therefore, the increase in porosity in our engineered wood could originate from the hygroscopic properties of callose. In addition, the reduced lignin content observed in our callose-enriched material may further enhance this increase in porosity^60^. Finally, the altered pore size in callose-enriched biomass (4–32 nm) coincides with the size of cellulase enzymes^61^ or some lytic polysaccharide monooxygenases^62^, consistent with the idea of improved enzymatic accessibility manifesting in the observed increase in saccharification yield (Fig. 5c, right).

### Biotechnological implications of callose-enriched LB

The results show that integrating callose into eudicot woody biomass can effectively manipulate wood crystallinity, mesoporosity and hygroscopicity—features known to be associated with increased hydrolysis of LB^63^. We hypothesize that callose integration into wood acts as a semi-permeable hydrophilic cell wall spacer increasing biomass porosity but reducing permeability, subsequently hindering lignin polymerization. Thus, the impact(s) on biomass accessibility may originate from a synergy of effects at different levels acting on the ultrastructure of secondary cell walls (porosity), its physico-chemical properties (crystallinity, hygroscopicity) and biochemistry (lignin synthesis). These effects in combination contribute to a substantial reduction in recalcitrance, perhaps due to improved access for hydrolytic enzymes and/or a more favourable environment (hydrophilic) for enzymatic activity. Beyond the digestion into monosaccharides, we foresee that improved enzymatic accessibility of our engineered wood will also benefit advanced biomaterials relying on LB deconstruction, such as cellulose nanofibrils, where cellulases and xylanases may be used^64^. Improved accessibility also has implications for treatment efficiency during the production processes of innovative wood-based biomaterials. Indeed, increasing wood porosity improves cell wall infiltration processes^65,66^. Finally, there is currently a growing interest to use wood as feedstock to fabricate cellulose-based advanced biomaterials through wood delignification to obtain cellulose scaffolds subsequently functionalized to specific applications^67–69^. We anticipate that the capacity to fine tune cell wall ultrastructure through genetic engineering will not only facilitate delignification processes, but also increase their functionalization capacity through improved LB accessibility. Furthermore, as integration of an ectopic polymer does not target the existing constituents of the modified biomass, we believe that our approach offers strong potential for stacking of biomass genetic modifications, which could include integration of more exotic polymers to pre-functionalize LB for specific properties and applications. In conclusion, our innovation defines a new way of thinking about the future of biomass engineering. Through integration of new polymers with various biochemical properties, we envision tailoring of biomass ultrastructure and properties for improved accessibility and physico-chemical features of interest.

## Methods

### Plant materials and growth conditions

*A. thaliana* lines used in this study were produced in Columbia 0 background. Growth chamber conditions for genetic transformation and phenotyping were as follows: 20 °C, 170 μmol m^−2^ s^−1^ photosynthetically active radiation (16 h light/8 h dark). In vitro hybrid aspen (*Populus tremula* × *tremuloides*) clone T89 (WT) and related transformants were clonally propagated and grown in a medium containing 0.22% MS basal salts plus vitamins (Duchefa, M0222), 1% sucrose and 0.7% Difco agar (BD Bioscience, BD 281230) at pH 5.6. After autoclaving, sterile filtered indole-3-acetic acid (Merck, I2886) was added to a final concentration of 1 mg l^−1^. To perform induction of the *cals3m* construct, β-17 estradiol (Merck, E1024, 5 µM final concentration) or DMSO (mock control) was added to the medium. In vitro saplings were grown at 20 °C with 85 μmol m^−2^ s^−1^ photosynthetically active radiation (16 h light/8 h dark) in sterile conditions. For solid-state NMR experiments, poplar shoot cuttings were allowed to root in the above-described medium for 2 weeks before being transferred to a medium where sucrose was replaced by d-glucose-^13^C_6_ (Merck, 389374) with no supplemented indole-3-acetic acid to the medium. After transfer into the d-Gglucose-^13^C_6_ supplemented medium, saplings were grown in a ^13^CO_2_ atmosphere^36,70^ for 10–14 weeks to allow consistent secondary growth in sapling stems (50% humidity, 24 °C, 500 ppm ^13^CO_2_). For greenhouse poplar phenotyping experiments, poplar saplings were cultivated in vitro under sterile conditions for 5–8 weeks, then transferred to soil (peat:sand:vermiculite mix in a 6:2:1 ratio) with Levington high nutrient M3, adapted for 2 weeks in contained small greenhouses (in growth chambers or directly in the greenhouse) and finally grown under normal greenhouse conditions (22–25 °C,18 h light) with automatic watering for another 12–14 weeks. For lignin analysis from hydroponics-grown trees, in vitro poplar shoots were allowed to root in the above-described medium (supplemented with no additive, DMSO or β-17 estradiol) for 4 weeks after propagation, before being transferred individually into glass jars containing rockwool and 200 ml of Hoagland’s solution (Sigma, H2395; supplemented with no additive, DMSO or β-17 estradiol) and allowed to grow 8 more weeks before sampling. For linkage analysis, thermoporosimetry (DSC) and saccharification experiments of in vitro-grown poplars, in vitro poplar shoots were allowed to root in the above-described medium (supplemented with no additive, DMSO or β-17 estradiol) for 4 weeks after propagation, before being transferred individually into glass jars containing 100 ml of fresh in vitro media with their original supplement (no additive, DMSO or β-17 estradiol) and allowed to grow for 6–8 more weeks before sampling.

### DNA constructs and transgenic plants

The promoters of *IRX3* (At5G17420), *IRX8* (At5G54690) and *ZCP4* (*Zinnia elegans ZCP4* gene for cysteine protease, GenBank: AB264052.1 (ref. ^27^)) genes were amplified by PCR and cloned using MultiSite Gateway (see Table 1 for primer list). For the *pAtIRX8-XVE::cals3m* estradiol-inducible construct, the promoters of IRX8 were amplified by PCR and cloned into P4P1RpGEMt containing the oestrogen receptor XVE^31,32^ by restriction ligation at the KpnI restriction site. Using the MultiSite Gateway system (Thermo Fisher), the promoters and their inducible versions were combined with the *cals3m* construct and the nopaline synthase terminator in destination vector pBm43GW or pHm43GW to produce the *pAtIRX3::cals3m*, *pAtIRX8::cals3m*, *pZeZCP4::cals3m* and *pAtIRX8-XVE::cals3m* constructs. *Arabidopsis* Col-0 plants were dipped with the different constructs and positive transformants were selected using phosphinothricin or hygromycin. Lines with single insertions were selected in T_2_ and homozygous plants were obtained in T_3_. Hybrid aspen (*Populus tremula* × *tremuloides*) clone T89 (WT) was transformed with the *pAtIRX8::cals3m* and *pAtIRX8-XVE::cals3m* constructs following ref. ^29^. For each of these constructs, we selected 5 lines out of 20 independent transformation events on the basis of the high intensity of their callose immunolocalization profile.

**Table 1:** List of primers used

Gene ID	Gene name	Primer name	Sequence	Orientation	Amplicon size (bp)	Fragment	pDONR^a^	Resistance
*At5G17420*	*IRX3*	MB044	ggggacaactttgtatagaaaagttgaaaaataagtaaaagatcttttag	fwd	1,172	promoter	p4p1z	Zeo
		MB045	ggggactgcttttttgtacaaacttgattagcagcgatcttgagagaac	rev				
*At2g23560*	*IRX8*	MB046	ggggacaactttgtatagaaaagttgacgagctgacttgtgtcgatgagc	fwd	1,485	promoter	p4p1z	Zeo
		MB047	ggggactgcttttttgtacaaacttgcgaagagggaaactggatcttacg	rev				
AB264052.1	*ZCP4*	MB048	ggggacaactttgtatagaaaagttgctcaagacatttcttacttatagac	fwd	1,854	promoter	p4p1z	Zeo
		MB049	ggggactgcttttttgtacaaacttgtgttgttgttgttgtggatgatg	rev				
*At2g23560*	*IRX8*	MB046	ccggtggtaccacgagctgacttgtgtcgatgagc	fwd	1,455	promoter	RpGEMt_P4P1_ml-XVE	Amp
		MB047	tacgtactcgagcgaagagggaaactggatcttacg	rev				

^a^pDONR is designing the type of Gateway™ entry vector used to clone the respective promoters.

### Histological analysis and immunolocalizations

Sections of *Arabidopsis* or poplar stems (3–5 mm long) were dissected with razor blades and immediately fixed in a solution consisting of 4% formaldehyde (freshly prepared from paraformaldehyde powder, Merck, 158127) and 0.5% glutaraldehyde (Merck) in PBS buffer. Fixation, dehydration, resin infiltration and antibodies washing steps were all microwave (MW) assisted using a PELCO BioWave Pro (Ted Pella). Fixation was realized at 150 W under vacuum (20 inHg, 5× 1 min). Samples were left in the fixative overnight at 4 °C and then washed 3 times in PBS. Depending on their sizes, stems were aligned in parallel and embedded in 1% agarose in PBS to include multiple biological replicates in the same resin block. Samples were then processed through increasing dehydration steps (25%, 50%, 70%, 90%, 96%, 3× 100% ethanol, vacuum 20 inHg, MW 150 W, 5 min) and left for at least 16 h in 100% ethanol at 4 °C. Resin infiltration (LR White medium grade, Agar Scientific) was then realized through increasing resin concentration: 33% resin in 100% ethanol, 66% resin in 100% ethanol and 3 times 100% resin (20 inHg, MW 200 W, 5 min). Samples were left for at least 24 h in 100% resin for effective penetration in the samples. Resin polymerization was subsequently realized at 60 °C for 18 h. Semi-thin sections (1 µm) were then obtained with a Leica EM UC7 ultramicrotome and laid on SuperFrost Ultra Plus microscopy slides (Thermo Fisher). Callose immunolocalizations on semi-thin sections were MW assisted and performed as follows: blocking step (2% BSA in PBS, 1 ml per slide, 30 min at r.t.); primary antibody (anti (1→3)-β-glucan (Biosupplies), 1/400 in 2% BSA in PBS, 500 µl per slide, ON 4 °C; 3 washes in 2% BSA in PBS (MW 170 W, 1 min)); secondary antibody (Alexa Fluor 488 anti-mouse IgG, Thermo Fisher, A-11017, 1/800 in 2% BSA in PBS, 500 µl per slide, 2 h at r.t.); 3 washes in 2% BSA in PBS (MW 170 W, 1 min). Slides were finally mounted in a 1:1 solution of AF1 antifadent (Citifluor) with PBS, containing calcofluor as a cell wall counterstain and imaged by confocal laser scanning microscopy (Zeiss LSM700 with Zen Black edition software v.14.0.27.201).

For immunolocalizations longitudinal to wood fibres, fixed wood samples (greenhouse or in vitro grown) were hand dissected with razor blades longitudinally to the stems and callose immunolocalizations were performed in tubes with gentle agitation as follows: 3 washes in PBS, blocking step: 2% BSA in PBS, 1 ml per slide, 1 h at r.t.; primary antibody (anti (1→3)-β-glucan (Biosupplies, 400-2)), 1/400 in 2% BSA in PBS, 500 µl per tube, ON 4 °C; 3 washes in 2% BSA in PBS; secondary antibody (Alexa Fluor 488 anti-mouse IgG, Thermo Fisher, A-11017) 1/800 in 2% BSA in PBS, 500 µl per slide, ON 4 °C; 3 washes in 2% BSA in PBS. Samples were then left in tubes containing a 1:1 solution of AF1 antifadent (Citifluor) with PBS, containing 0.1% (w/v) Direct Red 23 (Sigma 212490) and left ON at 4 °C under gentle agitation before being imaged by confocal laser scanning microscopy (Zeiss LSM700 with Zen Black edition software v.14.0.27.201).

For TEM immunolocalizations, 80–100-nm thin sections were obtained with a Leica EM UC7 ultramicrotome using an Ultra 45° diamond knife (DiATOME) and laid on 200-mesh formvar-coated Nickel grids (EM Resolutions, F200Ni100). Sections were blocked with filtered 2% BSA in PBS at r.t., labelled with mouse anti-callose IgG1 (anti-(1→3)-β-glucan; 1:10; Biosupplies, 400-2) ON at 4 °C, washed 6 times with 2% BSA in PBS (2 min each), incubated for 2 h at r.t. with donkey anti-mouse IgG coupled to 18 nm colloidal gold (1:30, Abcam), washed again 6 times with 2% BSA in PBS (2 min each), washed 3 times with water and post-stained for 5 min with uranyl acetate in 50% methanol before observation. Images were acquired with a Tecnai G2 80-200 keV transmission electron microscope run at 200 keV using a 20 µm objective aperture for contrast, an ORCA high-resolution CCD camera and the Image Capture Engine software (v.600.323, Advanced Microscopy Techniques).

### Image analysis and treatment

Fluorescence analysis of callose immunolocalizations was performed using a custom-made macro on ImageJ (v.1.53q). The images were acquired as *z*-stacks, then processed to maximal projections. The sequential steps of the macro were as follows: a region of interest restricted to the wood area was first hand drawn by the user; within the defined wood region of interest, a binary mask was applied with a user-defined threshold to the cell wall counterstaining channel (here calcofluor) to create a new region of interest restricted to the secondary cell area (the creation of a region of interest was restricted to the cell wall to avoid underestimating the levels of fluorescence measured in different conditions, as the studied epitopes are exclusively localized to the cell wall); this cell wall-specific region of interest was finally applied to the immunolocalized channel (here callose) to measure the average fluorescence levels. The detailed macro code can be found in Supplementary Document 1. To display the fine callose deposition patterns in longitudinal pictures in Fig. 4, immunolocalizations, acquired as *z*-stacks, were deconvoluted with Huygens Essential v.22.04 software using the recommended parameters of the deconvolution wizard. The deconvoluted *z*-stacks were then projected as maximal projections for display.

For vessel size and fibre size measurement, we used either the magic wand tool of ImageJ (v.1.53t) to define vessel regions of interest before measurement, or the software LithoGraphX v.1.2.2 with Builder v.1.2.2.7 (https://sourceforge.net/projects/lithographx/) to analyse maximal projections of calcofluor-stained sections following a previously described protocol^71^ with modification. Images were pre-processed in Fiji (v.1.0)/ImageJ (v.1.47) to meet the requirement of LithoGraphX analysis.

### *Arabidopsis* and poplar cell wall extracts preparation

Fresh plant material was snap frozen in liquid nitrogen and immediately ball milled in stainless steel 25-ml grinding jars (Retsch) for 1 min at 30 Hz. Ground material was subsequently collected in 15 ml falcon tubes and cell wall material was extracted as follows: chloroform:methanol (2:1) 1 h, 70% ethanol 1.5 h (×2), 80% ethanol 1 h, 95% ethanol 2 h, acetone 1 min. Each extraction step was performed with gentle agitation, followed by centrifugation at 4,500 *g* at 4 °C for 5 min. The resulting alcohol insoluble residues were then dried overnight under fume hood air flow and subsequently destarched twice as follows: 10 ml 2.5 U ml^−1^ α-amylase (type VI-B from porcine pancreas, Merck, A3176) in PBS 1X buffer (pH 7), incubated overnight to 24 h at 37 °C. Enzyme solution of the destarching steps was removed by centrifugation (4,500 *g*, 10 min, 4 °C). Residual pellets were finally washed as follows: 70% ethanol (×3), acetone (×3). Each washing step was done by manual shaking (1 min) and solvent removal was performed after centrifugation (4,500 *g*, 10 min, 4 °C). The resulting cell wall extracts (CWE) were finally dried overnight at room temperature under fume hood air flow.

### PACE for callose detection and xylan glucuronidation assessment in poplar biomass

Poplar CWE (2 mg) were pretreated with 4 M NaOH at room temperature for 30 min. The samples were then neutralized with HCl and diluted in 0.1 M ammonium acetate buffer (pH 5.5) to a concentration of 2 mg CWE ml^−1^. The material was digested for 24 h with *Hordeum vulgare* GH17 endo-1,3-β-glucanase (Megazyme) at 30 °C. For each digestion, 12.5 units of enzyme were used. Released oligosaccharides were dried and derivatized with 8-aminonapthalene-1,3,6-trisulphonic acid (ANTS, Invitrogen) as previously described^72^. PACE was on a gel with a continuous 10% acrylamide concentration run for 45 min at 1,000 V and visualized as previously described^72,73^. Digestion products released from poplar CWE were analysed alongside those produced from a callose polysaccharide standard (Biosupplies, 300-2) and an oligosaccharide standard on a Gbox equipped with Genesnap software v.7.12 (Syngene). All oligosaccharides were purchased from Megazyme and derivatized with ANTS. To assess xylan glucuronidation levels, 1 mg of poplar CWE was extracted with 4 M NaOH (20 µl) for 1 h. The extraction was neutralized with 1 M HCl and the biomass was resuspended to a final concentration of 1 mg ml^−1^ in 0.1 M ammonium acetate buffer (pH 5.5). For each digestion, 0.1 mg of extracted biomass was used. Xylooligosaccharides were released using xylanase GH11 from *Neocallimastix patriciarum* (Megazyme, 10 U used per digestion, incubation at 30 °C overnight). Following digestion, the oligosaccharides were analysed with PACE as previously described^48,73,74^ on a Chemidoc MP imaging system equipped with Image Lab touch (v.2.4.0.03, BioRad). PACE band intensity analysis was performed using ImageJ and the degree of glucuronidation was quantified as previously described^75^.

### Monosaccharide analysis

The monosaccharide composition was determined using two-step sulfuric hydrolysis followed by high-performance anion-exchange chromatography with pulsed amperometric detection (HPAEC-PAD) analysis, according to a previously reported procedure^76^. In brief, 1–2 mg CWE (triplicate) was hydrolysed using 72% H_2_SO_4_ for 3 h at room temperature, followed by a second hydrolysis step with 1 M H_2_SO_4_ for 3 h at 100 °C. The hydrolysed samples were then filtered through a Chromacol 0.2 μm filter (Thermo Fisher), diluted with Milli-Q water and analysed on an ICS3000 system (Dionex) with a Dionex CarboPac PA1 column at 30 °C, using the same elution programme as previously described^77^ and Chromeleon software v.7 (Dionex, Thermo Fisher). Quantification for HPAEC-PAD analysis was performed by external calibration using standard solutions containing different neutral monosaccharides (fucose, rhamnose, xylose, arabinose, glucose, mannose and galactose).

### Gycosidic linkage analysis

Glycosidic linkage analysis was performed in triplicate as previously described^78^ with modifications. A total of 1 mg CWE was dissolved in 400 μl of DMSO containing 0.3 mg l^−1^ sulfur dioxide (SO_2_) and 10 μl diethylamine, flushed with argon and stirred overnight at room temperature. The samples were then mixed with freshly ground NaOH (200 mg), flushed with argon and stabilized under stirring for 30 min. Methylation was performed by sequential addition (5×) of 30 μl of methyl iodide at 10 min intervals, with argon flushing and sonication in between each addition. The methylated polysaccharides were recovered in the organic phase after partition (3×) using H_2_O and dichloromethane and further dried under air. The methylated polysaccharides were hydrolysed with 1 ml of 2 M trifluoroacetic acid at 121 °C for 3 h and further dried under air. After hydrolysis, the released sugars were reduced with sodium borodeuteride in 1 M ammonia solution at room temperature for 1.5 h, quenched and dried (3×) with 10% acetic acid in methanol and further washed and dried (3×) with pure methanol. For derivatization, the samples were acetylated with pyridine and acetic anhydride (1:1 v/v, 200 μl) at 100 °C for 60 min. The per-*O*-methylated alditol acetates (PMAAs) were then extracted with ethyl acetate and quantified using an HP-6890 gas chromatograph and an HP-5973 electron-impact mass spectrometer fitted with an SP 2380 capillary column, using ChemStation D03 software (Agilent). The programme used was initially set to 163 °C, followed by ramping at 1 °C min^−1^ until 213 °C, ramping at 3 °C min^−1^ until 230 °C, ramping at 10 °C min^−1^ until 260 °C and holding for 10 min. The PMAAs were identified by their retention times and electron-impact profiles in comparison to available polysaccharide standards.

### Glycome profiling/ELISA experiments

Glycome analysis is an ELISA-based technique for polysaccharide detection on cell wall fractions. Samples of CWEs were sequentially extracted with cyclohexane diamine tetraacetic acid (CDTA), potassium hydroxyde (KOH) and cellulase as follows. CWE (4 mg) was ground using ball bearings at 50 Hz for 90 s. CDTA (1.5 ml, 50 mM) was added and samples were further ground for 10 min at 50 Hz, followed by rocking at room temperature for 50 min. Samples were then centrifuged at 3,500 *g* for 15 min, the supernatant retained as CDTA fraction, the residues washed twice with distilled water and then extracted in the same manner using 4 M KOH with 1% (w/v) sodium borohydride to give the KOH fraction. Washed residues were then treated with 1.5 ml 1 µg ml^−1^ cellulase (Cellulase 5A, NZYTech) in 20 mM Tris-HCl buffer (pH 8.8), rocked gently at 37 °C for 2 h before centrifuging at 3,500 *g* for 15 min and the supernatant retained as the cellulase fraction. A volume of 1.4 ml of all samples was adjusted to pH 7 using 60% (v/v) acetic acid and made up to 10 ml with 1× PBS buffer before coating 100 µl per well onto immunosorbent plates (Nunc MaxiSorp) and incubating at 4 °C for 16 h. The plates were washed 10× with tap water, patted dry and then blocked with 5% (w/v) dry milk powder (Marvel Milk) in 1× PBS buffer (MP/PBS) for 2 h at 200 µl per well. Following washing (10× tap water), plates were incubated with primary antibody diluted in MP/PBS for 1.5 h. All antibodies were used at 1:10 dilution, except for the callose antibody (Biosupplies, 400-2), which was purified (not hybridoma supernatant) and used at a 1/150 dilution. After washing, the plates were incubated with secondary antibody in PBS/MP at a 1/1,000 dilution for 1.5 h. Anti-rat IgG-HRP was used as the secondary antibody for all the antibodies, except for the callose antibody, which was raised in mouse; hence anti-mouse IgG-HRP was used. The plates were washed, followed by the addition of the substrate to generate the signal at 100 µl per well. The substrate contained 1 M sodium acetate buffer (pH 6.0), tetramethylbenzidine, 6% (v/v) hydrogen peroxide and distilled water at a ratio of 100:10:1:1,000. The reaction was stopped after 6 min by the addition of 50 µl per well of 2.5 M sulfuric acid and the binding strength of each antibody was determined by the absorbance at 450 nm using an ELISA plate reader (Multiskan Fc microplate readers, with SkanIt reader software from Thermo Fisher).

### RAMAN analysis

Three-month-old greenhouse- or in vitro-grown tree stems were used for this analysis. Basal cross sections were harvested and immediately fixed and stored in formaldehyde/acetic acid until use. Samples were thoroughly washed and mounted in water before RAMAN spectroscopy. Greenhouse-grown tree samples were hand sectioned while in vitro-grown trees were previously encased with 5% agarose to facilitate multiple samples sectioning with a Leica VT1200S vibratome. Raman microscopy was carried out on a Renishaw InVia instrument fitted with a 532 nm diode laser. Stem sections were mounted in water with a coverslip carefully placed on top and sealed with vacuum grease. A Leica HC PL APO ×20/0.75 IMM CORR CS2 objective was used with the collar set to water immersion. Raman acquisitions used a 2,400 l mm^−1^ grating, 1,400 cm^−1^ centre, 50% laser power (<15 mW at sample plane), pinhole engaged, regular confocal mode, 20 s pre-acquisition bleach and 0.3 s exposure with 15 accumulations. A total of 24 secondary walls were sampled for each section and then averaged using the ‘average collected datasets’ tool of Renishaw WiRE software v.4 to improve signal-to-noise ratio.

### Lignin acetyl bromide analysis

For lignin analysis, dried debarked wood samples from trees grown on soil or in hydroponics conditions were used. The lignin content was quantified by spectrophotometry using bvda software v.VA1.176 (VWR), following the acetyl bromide method^79^ using an exact mass of 20 mg of sample per assay. Samples were ground in liquid nitrogen for homogeneity before analysis. The chemicals were laboratory grade from Sigma Aldrich and the analyses were performed as three independent replicates, with the lignin content expressed as a percentage of dry matter.

### Solid-state NMR experiments

Poplar sapling bottom stem parts were snap frozen in liquid nitrogen, immediately reduced into powder and packed into a 3.2 mm MAS NMR rotor. One biological replicate from two independent *pAtIRX8-XVE::cals3m* lines (inducible lines), following estradiol induction, was used for ssNMR experiments and compared to their respective DMSO mock controls. Solid-state MAS NMR experiments were performed on a Bruker 850 MHz AVANCE NEO solid-state NMR spectrometer operating at ^1^H and ^13^C Larmor frequencies of 850.2 and 213.8 MHz, respectively, using 3.2 mm double-resonance MAS probes. Experiments were conducted at room temperature at MAS frequencies of 12.5 kHz unless otherwise stated. The ^13^C chemical shift was determined using the carbonyl peak at 177.8 ppm of l-alanine as an external reference with respect to tetramethylsilane; 90° pulse lengths were typically 3 µs (^1^H) and 3.3 µs (^13^C). Both ^1^H-^13^C CP with ramped (70–100%) ^1^H radiofrequency amplitude and a contact time of 1 ms and direct polarization (DP) were used to obtain the initial transverse magnetization^80^. While CP emphasizes the more rigid material, a short 2 s recycle delay DP experiment was used to preferentially detect the mobile components and a 20 s delay was used for quantitative experiments. SPINAL-64 decoupling was applied during acquisition at a ^1^H nutation frequency of 80 kHz (ref. ^81^). The proximity of water to different components of the plant was studied using a water-edited CP experiment^42,43^. The CP parameters were as stated above, with the proton filter time being 3 ms and the diffusion time varying from 1 to 25 ms for both samples. Intermolecular contacts were probed using 2D ^13^C-^13^C ^1^H driven spin diffusion (PDSD) experiments with mixing times of 30 ms to 1.5 s (ref. ^82^). The acquisition time in the indirect dimension (*t*_1_) of the CP PDSD experiments was 6–7 ms. The sweep width in the indirect dimension was 50 kHz with 48 acquisitions per *t*_1_ for both CP PDSD experiments and the recycle delay was 2 s. For the PDSD experiments, the spectra were obtained by Fourier transformation into 4 K (F_2_) × 2 K (F_1_) points with exponential line broadening of 50 Hz in F_2_ and squared sine bell processing in F_1_. All spectra obtained were processed and analysed using Bruker Topspin v.3.2.

### Water vapour sorption measurement

Water vapour sorption and desorption measurements were performed at 25 °C using a regulated atmosphere Cahn D200 microbalance equipped with DVS-Advantage control software v.2.1.5.1 (Dynamic Vapour Sorption from Surface Measurement Systems) with a mass resolution of 0.1 µg. The DVS apparatus allows collection of kinetic data by recording mass evolution vs time as a function of a given water activity. The initial mass of dried samples was ~20 mg. After exposure to dry nitrogen flux until a constant sample mass was reached (*m*_d_), water vapour was flushed at controlled pressure and the mass uptake was measured as a function of time. For this, successive water activity steps ranging from 0.1 to 0.9 by steps of 0.1 were performed. The sorption kinetics were followed step by step until equilibrium. For the desorption kinetics, the relative humidity steps were decreased to 0 in a reverse order to measure the equilibrium water contents as a function of time. The sorption or desorption equilibrium for each activity was reached as the change in mass with time was below a predefined threshold value. Water activity *a*_w_ was adjusted by mixing dry and saturated nitrogen gases using electronic mass flow controllers. The water mass gain *M* at sorption equilibrium (expressed in percentage or gram of water per 100 g of sample) was calculated for each water vapour activity as:

$$M=\frac{{m}_{{\rm{eq}}}-{m}_{\rm{d}}}{{m}_{\rm{d}}}\times 100$$

where *m*_d_ and *m*_eq_ are the dry mass and the mass at equilibrium state of a sample, respectively. Likewise, the equilibrium water contents at each water vapour activity for the desorption stage were calculated. Water vapour sorption and desorption isotherms were thereafter determined from kinetic data by plotting the water mass contents at equilibrium at each water activity step. The accuracy of mass gain values at equilibrium was estimated to be better than 2%. The Park model was used to correlate model parameters with physical characteristics^83^. This model is expressed as:

$$M=\frac{{A}_{\rm{L}}{b}_{\rm{L}}{a}_{\rm{w}}}{1+{b}_{\rm{L}}{a}_{\rm{w}}}+{k}_{\rm{H}}{a}_{\rm{w}}+n{k}_{\rm{H}}^{n}{k}_{a}{a}_{\rm{w}}^{n}$$

where ${A}_{\rm{L}}$ is the Langmuir capacity constant, ${b}_{\rm{L}}$ is the Langmuir affinity constant, ${a}_{\rm{w}}$ is the water activity, ${k}_{\rm{H}}$ is Henry’s solubility coefficient, ${k}_{a}$ is the equilibrium constant for the clustering reaction and $n$ is the mean number of water molecules per cluster.

To evaluate the goodness of fit of isotherm curves, the mean relative percentage of deviation modulus, noted as MRD, was calculated as:

$${\rm{MRD}}=\frac{100}{N}\times \mathop{\sum }\limits_{i=1}^{N}\frac{\left|{m}_{i}-{m}_{{pi}}\right|}{{m}_{i}}$$

where ${m}_{i}$ is the experimental value, ${m}_{{pi}}$ is the predicted value and $N$ is the number of experimental points. The MRD is widely adopted in the literature and a modulus value below 10% indicates a good fit^84^.

### Thermoporosimetry

Water thermoporosimetry is based on the Gibbs–Thomson effect where water crystals located in porous structure require more energy to melt due to the pore surface energy.

When approximated to perfect cylinders, the diameter of the pores can be calculated from their melting point on the basis of the equation (1)^85–87^.

1 $${D}=4\,T_0\,{\gamma}\,{\rm{cos }}({\theta })/((T_{\rm{m}}-T_0)\,{\rho }\,{\rm{Hf}})$$

where *D* is the diameter of the pore in metre, *T*_m_ is the depressed freezing temperature in Kelvin, *T*_0_ = 273.15 K, *γ* = 12.1 mJ m^−2^, *θ* = 180°, *ρ* = 1,000 kg m^−3^ and Hf = 334 J g^−^^1^.

Thermoporosimetry using DSC is based on a series of arbitrarily chosen isothermal steps^45,86,88^. The water melting enthalpy occurring at each of the isotherm steps is used to calculate the portion of water retained in the pore of a calculated diameter range. For this experiment, in vitro poplar saplings were sampled as follows: 3 cm from the basal part of the stems were dissected with razor blades, immediately debarked, immersed in a solution of 0.1% sodium azide (Merck, 71290) and kept at 4 °C until use. Before analysis, the debarked stems were dissected again with razor blades and thermal analysis of the stems was carried out on a Discovery DSC using Tzero hermetic aluminum pans (TA instrument). Around 5–10 mg of sample was cooled from room temperature to −30 °C following the sequence presented in Supplementary Table 3. Experiments were carried out in triplicates and results were interpreted with TA Trios v.5.1.1.46572. Samples were soaked for 30 min in Milli-Q water, then lightly dabbed with a cotton fabric before being sealed in the DSC hermetic pans. The total water content of the fully saturated sample was measured by puncturing the DSC pans then placing them in an oven at 103 °C for at least 2 h.

### Biomass crystallinity

At 3 months after transfer to soil, greenhouse-grown wood samples were harvested from saplings, debarked and dried overnight in an oven at 55 °C. Crystallinity was determined on eight individuals per line using a Bruker D8 Discover wide angle X-ray diffraction instrument equipped with an area detector (GADDS). The measurements were collected in transmission mode using CuKα radiation source emitting at 0.154 nm wavelength, with X-ray optics *θ*1 = 17° (*θ*1 = source, *θ*2 = detector). Diffraction intensities were collected at Bragg angles 2*θ* = 4°−40. The X-ray diffraction profile was integrated at Chi between −180° and 0° using GADDS software (Bruker AXS). The data were normalized and resolved using the crystallinity calculation method of Vonk^89^. First, the background diffraction signal was subtracted, then the amorphous curve was fitted to the diffraction pattern and finally, linear regression analysis was conducted to obtain cell wall crystallinity.

### Tensile properties

The basal parts of 3-month-old stems (biological replicates) were sectioned into ~3-cm-long pieces using a hand saw. Afterwards, these stem sections were stored in water for >24 h. Using a rotary microtome (Leica RM2255), 100-μm-thin longitudinal-tangential cuts (technical replicates) were prepared in the wet state until the pith was reached. The cuts were numbered from the outside towards the centre (pith) and dried again under ambient conditions. For the microtensile testing, 10 technical replicates of 100-μm thickness (evenly distributed from the outside towards the centre) were stored in water for >1 h before an ~2-mm-wide strip was cut from the centre of the cut. The thickness of the wet samples was determined in three different positions using a micrometre. The samples were placed in the microtensile testing setup, equipped with a 50 N load cell (Honeywell Model 31 50N) and a microscope (Leica Z6 APO equipped with a Basler scA1390-17gm digital camera) to track displacement and determine the width of the specimen and the total clamping distance (again at three different positions). The clamping distance was set to ~1 cm, the test speed to 10 μm s^−1^ and the specimens continuously moistened using a pipette during the test to avoid drying. The loading conditions and data collection were performed via a Labview (v.10.0) interface. In total, ten technical replicates for each of the ten biological replicates of WT, Const. L11 and Const. L17 were tested.

### Green density

The green density $\left(R=\frac{{m}_{{\rm{dry}}}}{{V}_{{\rm{wet}}}}\right)$ was analysed across the stem radius for ten evenly distributed cuts for three biological replicates (the ones with the highest and lowest tensile strength and from in-between) of WT, Const. L11 and Const. L17. The cuts were placed in water for >1 h and an ~2-mm-wide and 1.5-cm-long piece was cut from the centre. To determine the wet volume, *V*_wet_, the thickness of the wet cut was analysed in three positions using a micrometre and the area was analysed using a microscope (Leica Z6 APO equipped with a Basler scA1390-17gm digital camera) and the image processing programme ImageJ (v.1.53k). The cuts were placed in an oven at 103 °C for >4 h and then the dry mass *m*_dry_ was evaluated using a precision scale (Mettler Toledo AE163).

### Cellulose MFA determination using wide-angle X-ray scattering

MFA determination was performed using a previously published protocol^90^ for ten biological replicates of WT, Const. L11 and Const. L17 (for each of three specimens). One specimen was taken from the periphery of the stem, one from the centre of the stem and one from a position in-between. Specimens were mounted on a sample holder and placed in the sample chamber of a laboratory SAXS system (Xenocs Xeuss 3.0). The 2D scattering signal was recorded on a 2D detector (Dectris EIGER2 R 1M) under vacuum, using CuKα X-ray radiation (*λ*_CuKα_ = 1.5419 Å) and a sample detector distance set to 50 mm. The total exposure time was set to 20 min with the line eraser setting turned on (records two diffraction patterns with a slightly shifted detector position and merges the two files to remove the slit in the detector, otherwise visible and impeding the radial integration). To obtain azimuthal intensity profiles, the 2D diffraction signal was then radially integrated around the 200 peak of cellulose, with an integration width of 0.22 Å^−1^. All data handling was performed using Xenocs XSACT software v.2.6.

Cell wall orientation was analysed for the cuts used in the wide-angle X-ray scattering experiment for three biological replicates of WT, Const. L11 and Const. L17. The cell wall orientation was then averaged for the three different positions (periphery of the stem, centre of the stem and in-between) of the cuts for each line and this average was used for the MFA determination. To obtain the cell wall orientation of the cuts, cross sections of the analysed cuts were prepared using a rotary microtome (Leica RM2255). Images were taken using an optical microscope (Olympus BX51 equipped with an Olympus SC50 digital camera) and the cell wall orientation was analysed using ImageJ (v.1.53k).

### Saccharification and SSF of poplar biomass

Saccharification experiments were performed as previously described^49^. In brief, poplar CWEs were resuspended in 0.1 M ammonium acetate buffer (pH 5.0) to a concentration of 1 mg ml^−1^. To this suspension, 20 µl of Cellic CTec2 was added for each 1 mg of plant material to be digested. Before the saccharification reaction, Cellic CTec2 was purified from residual sugars with PD-10 desalting columns (GE Life Sciences) and diluted by a dilution factor 10 in 0.1 M ammonium acetate buffer (pH 5.0). The saccharification reaction was performed in a thermomixer set to 45 °C. Samples were removed from the saccharification vessel at specific time points. The concentration of glucose and xylose in each sample was measured using enzymatic assays (Megazyme). Each measurement was standardized to that obtained for biomass only and enzyme only samples.

SSF experiments were performed as previously described^49^. In brief, 50 mg of poplar CWE was resuspended in 9.5 ml LB medium in a 15 ml falcon tube. The suspension was sterilized by incubation at 85 °C for 10 min, followed by cooling on ice. Thereafter, the mix was amended with 250 µl Cellic CTec2 solution, which was also used in the saccharification experiments, and with 250 µl *E. coli* inoculum. For this experiment, *E. coli* bearing the BBa_K1122676 BioBrick enabling isopropyl β-d-1-thiogalactopyranoside (IPTG) inducible ethanolic fermentation was used^50^. The optical density (OD)_600_ of the inoculum was within the 0.55–0.6 range for all experiments. The suspension was also amended with 10 µl of chloramphenicol solution (25 mg ml^−1^) and IPTG (1 M). The tube was sealed with parafilm and SSF was performed for 96 h in an incubator set to 37 °C and 200 r.p.m. Following incubation, ethanol concentration was determined in the supernatant of the culture using commercial ethanol assay (Megazyme, K-ETOH).

### Graphical and statistical analysis

Data analysis used the Tidyverse R package collection and the ggplot2 package for boxplots and bar plots. Statistical analysis was performed using the R functions for analysis of variance (ANOVA) and Tukey’s honestly significant difference tests for multiple comparisons after having determined that parametric tests were applicable using R functions for Bartlett and Shapiro tests. In the case where parametric tests could not be applied, statistical analysis was performed using the R functions for Kruskal–Wallis one-way ANOVA and a non-parametric test for multiple comparisons (nparcomp package). Significance values for *P* < 0.05 were grouped using the agricolae package (with alpha = 0.05).

### Reporting summary

Further information on research design is available in the Nature Portfolio Reporting Summary linked to this article.

## Supplementary information

Supplementary Information Supplementary Tables 1–3, Notes 1–3, Fig. 1, Document 1 and References.

Reporting Summary

## Data Availability

All data are available from the Cambridge Apollo Repository (10.17863/CAM.96886). Source data are provided with this paper.

## Source data

Source Data Fig. 1 Unprocessed PACE gel for Fig. 1m.
Source Data Extended Data Fig. 3 Unprocessed PACE gels for Extended Data Fig. 3d.
Source Data Extended Data Fig. 4 Statistical source data for Extended Data Fig. 4a,b.

## Code availability

The ImageJ macro code used to analyse callose immunofluorescence levels is available in Supplementary Information.
