# Supplementary material for: Ectopic callose deposition into woody biomass modulates the nano-architecture of macrofibrils
Source: Nat Plants. 2023 Sep 4;9(9):1530–46. doi: 10.1038/s41477-023-01459-0 (PMC10505557; doi:10.1038/s41477-023-01459-0)
Supplement: Supplementary file 1 — Supplementary Tables 1–3, Notes 1–3, Fig. 1, Document 1 and References. [file 41477_2023_1459_MOESM1_ESM.pdf]

nature plants

Article

https://doi.org/10.1038/s41477-023-01459-0

Ectopic callose deposition into woody biomass modulates the nano-architecture of macrofibrils

In the format provided by the authors and unedited

Supplementary information for Ectopic callose deposition into woody biomass modulates the nano-architecture of macrofibrils

Supplementary tables

	C1 / ppm	C2 / ppm	C3 / ppm	C4 / ppm	C5 / ppm	C6 / ppm
Callose in poplar A	104.5	73.4	87.0	69.1	75.8	61.4
Callose in poplar B	103.8	74.5	87.0	68.4	77.7	61.4
Hydrated Curdlan (Saito et al., 1990)	104.3	73.8	87.3	69.1	75.8	61.2
Annealed Curdlan (Saito et al., 1990)	103.5	74.3	86.5	68.3	77.5	61.6
Hydrated Curdlan (Pelosi et al. 2006)	104.4	73.0	86.6	68.7	76.1<br>75.5	61.1
Hydrothermally annealed Curdlan (Pelosi et al. 2006)	103.5	74.1	86.7	68.0	77.3<br>75.8	61.0

Supplementary table 1: ssNMR Carbon chemical shifts of callose compared to Curdlan.

The Carbon chemical shifts of the two callose environments (A and B) were deduced from the 1D ^{13}C CP MAS and 2D CP-PDSD experiments. They are here compared to previously chemical shifts measured from hydrate and annealed Curdlan from Saitô et al.[1] and Pelosi et al.[2]. The shifts determined by Saito et al. and Pelosi et.al are typically 0.2-0.3 ppm less than those we determined for callose in poplar. This systematic difference is likely due to their use of the glycine carboxyl as a ^{13}C reference whereas we used the L-alanine carboxyl.

sample	line	Shift (ppm)	Width (ppm)	%
Ind. L11 DMSO	Free water	4.72	0.09	25.5
Ind. L11 DMSO	Bound water	4.61	0.23	46.7
Ind. L11 DMSO	Background 1	4.75	0.70	21.6
Ind. L11 DMSO	Background 2	4.00	1.00	6.2
Ind. L11 Est.	Free water	4.72	0.10	76.8
Ind. L11 Est.	Bound water	4.61	0.23	8.0
Ind. L11 Est.	Background 1	4.84	0.40	12.9
Ind. L11 Est.	Background 2	4.00	1.00	2.4

Supplementary table 2: Fitting parameters used in the simulation of the ^{1}H spectra of poplar samples.

	Start temp	End temp	Isothermal at end temperature duration (min)	Ramp (°C/min)	Notes
1	-30	-20	5	1	Cp det
2	-20	-15	5	1	
3	-15	-10	5	1	
4	-10	-6	10	1	
5	-6	-4	10	1	
6	-4	-2	10	1	
7	-2	-1.5	15	1	
8	-1.5	-1.1	15	1	
9	-1.1	-0.8	15	1	
10	-0.8	-0.5	15	1	
11	-0.5	-0.2	15	1	
12	-0.2	-0.1	15	1	

Supplementary table 3: DSC thermoporosimetry subsequent ramp/isothermal steps.

Supplementary notes

Supplementary note 1: Choice of secondary cell wall promoter leads to different callose synthesis patterns in *Arabidopsis*.

These promoters have been chosen for their specificity in secondary cell wall synthesis: IRX8 is involved in glucuronoxylan and homogalacturonan synthesis and has been demonstrated to be mostly expressed in xylem and interfascicular fibers[3]; *AtIRX3* codes for the CESA7 subunit of the cellulose synthase complex (CSC) specifically involved in cellulose production in the secondary wall[4] and shows a strong expression in xylem and interfascicular fibers[5]; *ZeZCP4* codes for a cysteine protease in the model *Zinnia elegans* and its promoter has been associated with an expression in immature tracheary elements in *Arabidopsis thaliana*[3, 4, 6, 7]. We obtained several genetically transformed *Arabidopsis* lines showing no growth penalty using the *AtIRX8* and *ZeZCP4* promoters, but the use *AtIRX3* promoter resulted in shorter stems, despite no visible growth phenotype before the bolting stage (Extended Data Fig. 1a). We assessed the presence of callose by immunolocalization, and a positive signal proved callose synthesis in secondary xylem vessels and interfascicular fibers tissues when using the *AtIRX8* and *AtIRX3* promoters when compared to Col-0 control (Fig S1 b to e and h-i). When using the *ZeZCP4* promoter, callose deposition was restricted to the lumen side of xylem (Fig S1f, arrowheads), but absent from interfascicular fibers (Extended Data Fig. 1g), consistent with a late deposition of callose during vessel differentiation. In *Zinnia elegans*, *ZeZCP4* codes for a papain-like cysteine protease, whose transcript is transiently induced to high levels just before autolysis of tracheary elements cell death[8]. As programmed cell death (PCD) is associated with terminal xylem differentiation, this expression profile could explain the late callose deposition pattern restricted to the xylem lumen in *Arabidopsis*.

Supplementary note 2: Glycome profiling suggest a strong integration of callose into secondary cell walls.

Epitope profiling was performed following a combination of 3 sequential biochemical or enzymatic extraction steps (CDTA, KOH, cellulase), consecutively extracting pectin, hemicellulosic, and cellulosic fractions. No major nor significant changes were detected when comparing the callose poplars enriched lines relatively to their controls (constitutive lines minus WT and inducible lines minus DMSO and WT variations, Extended Data Fig. 5a). A slight increase in xylan and xyloglucan was detected in the constitutive lines in the KOH fraction (hemicellulosic fraction), which could suggest an accessibility increase to those polymers upon callose deposition. However, this effect wasn't detected in our inducible lines, what makes such assumption difficult to correlate directly to callose synthesis. An interesting observation can be made when extracting the callose data from these ELISA experiments. On the general glycome profiling (Extended Data Fig. 5a) and on an independent ELISA experiment targeting specifically callose detection (Extended Data Fig. 5b), callose was detected at highly significant levels in all 3 sequentially extracted cell wall fractions in all our lines, suggesting a strong integration of the ectopically deposited callose into secondary cell walls and a potential spatial proximity of callose to other cell wall polymers.

Supplementary note 3: Dynamic Vapor Sorption detailed analysis.

NB: for Park model parameters details, see methods section.

DVS is a gravimetric sorption technique that measures how quickly and how much of a solvent (in this case water) is absorbed by a sample. It measures the change of mass of a sample at increasing (sorption) or decreasing (desorption) relative humidity (RH) when reaching a mass plateau (called isotherm) at each RH measured point. DVS analysis usually display a classical type II sigmoidal profile dependent to the RH zone considered. The 0-20% RH zone is generally attributed to the sorption of a molecular monolayer of water, adsorbed on hydrophilic groups at the substrate surface, such as the internal surface of the cell wall. The 20-70% RH zone is related to the random sorption of water by dissolution and diffusion of water molecules inside the secondary wall and is referred to as the Henry-law sorption[9, 10]. This second sorption step depends not only on the accessible polar groups of polysaccharides but is also impacted by the pectic polyanions in the wood substrate. The absorbed water molecules are located within the space between cellulose microfibrils in the cell wall. This space is referred to as transient microcapillarity network where water molecules are linked to the OH groups by monolayer association through hydrogen bonds. The 70-100% RH zone reflects the clustering of water molecules at equilibrium state and is related to the meso-porosity of the assessed material. Note that a continual exchange is performed between monolayer and polylayer water molecules as a result of a non-static process. It should be noted that the sample masses were kept constant for the different experiments allowing comparison between samples. The tested humidity range did not exceed 90% RH (*i.e.* $a_w = 0.9$) to avoid further sorption occurring within macrovoids (such as cell wall lumina) in natural wood or fibers due to water condensation[11]. As the partial pressure is increased or decreased in steps, the water mass gain of the

sample changes accordingly with an asymptotic profile until a constant value is reached at equilibrium state. An example of run for a sorption and desorption cycle is shown in Supplementary Figure 1a. As seen, the mass gain gradually increased during the sorption process and decreased during the desorption process, as the target partial pressure of water started until the sorption or desorption reached the equilibrium state. At equilibrium state, the partial pressure changed to the next preset values. In Supplementary Figure 1b, the reproducibility of the sorption/desorption process for a WT sample is highlighted. The data of two distinct runs of the same sample are superimposable over the water activity range and the water vapor sorption/desorption isotherms exhibited the classical type II sigmoidal profile and hysteresis between sorption and desorption loops. Only a very small deviation in the data at very high-water activities is discernable and is usually related to the measurement accuracy of such device. The water vapor sorption isotherms were fitted by the Park model and the low values of the mean relative deviation modulus (below 10%) confirms the good fitting of the experimental points (Supplementary Figure 1c). An increase of the k_H coefficient and the *Ka* equilibrium constant is observed for the clustering reaction upon callose synthesis (Ind. L11 estradiol *versus* DMSO), in agreement with an increase in OH groups access in the samples. Accordingly, the mean number of water molecules per aggregate (n) is increased from 7 to 8 suggesting an increase in the meso-porosity of material.

Supplementary Figures

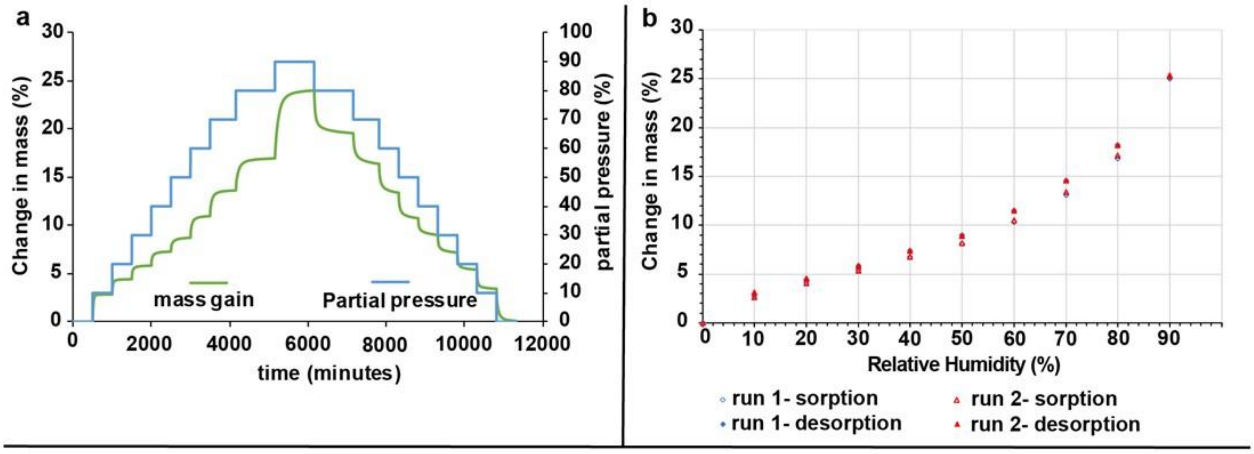

c

Park parameters						
Constants	A_L	b_L	K_H	Ka	n	MRD %
**Ind. L11, DMSO**	1.46	72.6	14.7	19.4	7	1.4
**Ind. L11, Estradiol**	1.89	34.6	15.6	35.0	8	1.9

Supplementary Figure 1: Examples of DVS kinetic and sorption curves on controls wood samples. DVS Park parameters of Ind. L11

a Kinetic curve from a sorption and desorption cycle applied to Ind. L11, DMSO (mock control) at 25°C. **b** Comparison of water sorption isotherms of two specimens of one control sample (WT). **c** sorption parameters of Park model determined from water vapor isotherms of the Ind. L11 samples in DMSO (mock) and callose inducing (estradiol) conditions.

Supplementary documents

Supplementary document 1: ImageJ analysis macro for callose immunolocalization fluorescence analysis.

```
"Stack to MAX, scale bar, save composite and individual channels, Define cell wall ROI from channel 1, save ROI and measure cell wall fluorescence in Channel 2 on defined ROI"
{
dir=getDirectory("Choose directory");
name=File.nameWithoutExtension;
print(dir);
AnalysisDir= dir+"/Analysis and splitted files/";
print(AnalysisDir);
File.makeDirectory(AnalysisDir);
path = AnalysisDir+name;
run("Z Project...", "projection=[Max Intensity]");
saveAs("Tiff", path+"_MAX_Tiff");
makeRectangle(1359, 120, 600, 24);
setTool("rectangle");
waitForUser("Move rectangle selection to adjust Scale bar position");
run("Scale Bar...", "width=250 height=30 font=100 color=White background=None location=[At Selection] bold overlay");
run("Make Composite");
saveAs("Jpeg", path+"_MAX_Composite");
imageTitle=getTitle();
run("Split Channels");
selectWindow("C1-"+imageTitle);
saveAs("Jpeg", path+"_MAX_C1");
setTool("polygon");
waitForUser("Draw manually ROI: Drawing inside current selection while pressing Alt creates a hole removing content from the ROI,\n Drawing outside current selection while pressing Shift creates new content")
run("Clear Outside", "stack");
setThreshold(27, 255);
run("Make Binary", "method=Li background=Dark calculate only black");
run("Create Selection");
roiManager("Add");
saveAs("Selection", path+"_ROI");
selectWindow("C2-"+imageTitle);
saveAs("Jpeg", path+"_MAX_C2");
roiManager("Select", 0);
waitForUser("Check that ROI match well the area to be measured");
run("Set Measurements...", "area mean standard min display redirect=None decimal=2");
run("Measure");
roiManager("Delete");
close("*")
}
```

Supplementary Information References

1. Saitô, H., Yoshioka, Y., Yokoi, M. & Yamada, J. Distinct gelation mechanism between linear and branched (1→ 3)-β-D-glucans as revealed by high-resolution solid-state 13C NMR. *Biopolymers: Original Research on Biomolecules* **29**, 1689-1698 (1990).
2. Pelosi, L., Bulone, V. & Heux, L. Polymorphism of curdlan and (1 → 3)-β-d-glucans synthesized in vitro: A 13C CP-MAS and X-ray diffraction analysis. *Carbohydrate Polymers* **66**, 199-207 (2006).
3. Persson, S. et al. The Arabidopsis irregular xylem8 mutant is deficient in glucuronoxylan and homogalacturonan, which are essential for secondary cell wall integrity. *The Plant Cell* **19**, 237-255 (2007).
4. Taylor, N.G., Scheible, W.-R., Cutler, S., Somerville, C.R. & Turner, S.R. The irregular xylem3 locus of Arabidopsis encodes a cellulose synthase required for secondary cell wall synthesis. *The plant cell* **11**, 769-779 (1999).
5. Mitsuda, N. et al. NAC Transcription Factors, NST1 and NST3, Are Key Regulators of the Formation of Secondary Walls in Woody Tissues of Arabidopsis. *The Plant Cell* **19**, 270-280 (2007).
6. Pyo, H., Demura, T. & Fukuda, H. Spatial and temporal tracing of vessel differentiation in young Arabidopsis seedlings by the expression of an immature tracheary element-specific promoter. *Plant and cell physiology* **45**, 1529-1536 (2004).
7. Pyo, H., Demura, T. & Fukuda, H. TERE; a novel cis-element responsible for a coordinated expression of genes related to programmed cell death and secondary wall formation during differentiation of tracheary elements. *The Plant Journal* **51**, 955-965 (2007).
8. Demura, T. et al. Visualization by comprehensive microarray analysis of gene expression programs during transdifferentiation of mesophyll cells into xylem cells. *Proceedings of the National Academy of Sciences* **99**, 15794-15799 (2002).
9. Sander, R. Compilation of Henry's law constants (version 4.0) for water as solvent. *Atmospheric Chemistry and Physics* **15**, 4399-4981 (2015).
10. Célino, A., Fréour, S., Jacquemin, F. & Casari, P. The hygroscopic behavior of plant fibers: a review. *Frontiers in chemistry* **1**, 43 (2014).
11. Hill, C.A., Norton, A. & Newman, G. The water vapor sorption behavior of natural fibers. *Journal of Applied Polymer Science* **112**, 1524-1537 (2009).
