# Supplementary material for: Ectopic callose deposition into woody biomass modulates the nano-architecture of macrofibrils
Source: Nat Plants. 2023 Sep 4;9(9):1530–46. doi: 10.1038/s41477-023-01459-0 (PMC10505557; doi:10.1038/s41477-023-01459-0)
Supplement: Supplementary file 2 — Reporting Summary [file 41477_2023_1459_MOESM2_ESM.pdf]

nature portfolio

Corresponding author(s): Matthieu Bourdon
Last updated by author(s): May 28, 2023

Reporting Summary

Nature Portfolio wishes to improve the reproducibility of the work that we publish. This form provides structure for consistency and transparency in reporting. For further information on Nature Portfolio policies, see our Editorial Policies and the Editorial Policy Checklist.

Statistics

For all statistical analyses, confirm that the following items are present in the figure legend, table legend, main text, or Methods section.

n/a	Confirmed	
☐	☒	The exact sample size (*n*) for each experimental group/condition, given as a discrete number and unit of measurement
☐	☒	A statement on whether measurements were taken from distinct samples or whether the same sample was measured repeatedly
☐	☒	The statistical test(s) used AND whether they are one- or two-sided<br>*Only common tests should be described solely by name; describe more complex techniques in the Methods section.*
☐	☒	A description of all covariates tested
☐	☒	A description of any assumptions or corrections, such as tests of normality and adjustment for multiple comparisons
☐	☒	A full description of the statistical parameters including central tendency (e.g. means) or other basic estimates (e.g. regression coefficient) AND variation (e.g. standard deviation) or associated estimates of uncertainty (e.g. confidence intervals)
☐	☒	For null hypothesis testing, the test statistic (e.g. *F*, *t*, *r*) with confidence intervals, effect sizes, degrees of freedom and *P* value noted<br>*Give P values as exact values whenever suitable.*
☒	☐	For Bayesian analysis, information on the choice of priors and Markov chain Monte Carlo settings
☒	☐	For hierarchical and complex designs, identification of the appropriate level for tests and full reporting of outcomes
☒	☐	Estimates of effect sizes (e.g. Cohen's *d*, Pearson's *r*), indicating how they were calculated

Our web collection on statistics for biologists contains articles on many of the points above.

Software and code

Policy information about availability of computer code

Data collection

Confocal images were acquired on a Zeiss LSM700 using the Zen Black v14.0.27.201 edition software.
TEM images were acquired on a Tecnai G2 80-200 keV transmission electron microscope using the Image Capture Engine software version 600.323 (Advanced Microscopy Techniques Corp, Danvers, USA).
PACE analysis were realized either with a Gbox equiped with Genesnap software v7. 12 (Syngene) or Chemidoc MP imaging system equiped with Image Lab touch software v2.4.0.03 (BioRad).
Ion chromatography data for monosaccharide analysis were acquired with Chromeleon software v7 (Dionex ThermoFisher scientific).
Gas chromatography data for linkage analysis were acquired with ChemStation D03 software(Agilent Technologies).
Glycome profiling and ELISA experiments data were collected using microplate readers equiped with the SkanIt reader software (ThermoFisher scientific).
RAMAN data were collected using the Renishaw WiRE software v4.
Lignin acetyl bromide analysis data were collected using a spectrophotometer equiped with bvda software version VA1.176 (VWR International).
Solid State NMR data were collected using the Bruker Topspin version 3.2 software.
Water vapor sorption analysis were collected a sorption analyzer equiped with DVS-Advantage control software v2.1.5.1 (Surface Measurement Systems).
Thermoporosimetry data were collected using the Trios software v5.1.1.46572.
Crystallinity was determined using a Bruker D8 Discover equiped with GADDS software (Bruker AXS Inc., Madison, WI).
Tensile Properties were collected using the Labview (Version 10.0) software.
Green density data were acquired with ImageJ software v1.53k.
Cellulose microfibril angle (MFA) data were collected with the Xenocs XSACT software version 2.6.

Data analysis	Confocal images were processed with ImageJ V1.47, V1.53k, V1.53t, and analyzed either with ImageJ V1.53k and V1.53t or the software LithoGraphX v.1.2.2 with the Builder v.1.2.2.7 (https://sourceforge.net/projects/lithographx/) for vessel and fiber size assessment. The ImageJ macro code used to analyze callose immunofluorescence levels is available in supplementary information. Confocal pictures used to describe callose deposition in longitudinal cell walls were deconvoluted with Huygens Essential V22.04.<br>ssNMR data were anayzed on Bruker Topspin version 3.2 software.<br>All other quantitative and statistical analysis were performed on RStudio version 2022.07.01 build 544, including the following packages: Tidyverse, ggplot2, agricolae, nparcomp.

For manuscripts utilizing custom algorithms or software that are central to the research but not yet described in published literature, software must be made available to editors and reviewers. We strongly encourage code deposition in a community repository (e.g. GitHub). See the Nature Portfolio guidelines for submitting code & software for further information.

Data

Policy information about availability of data

All manuscripts must include a data availability statement. This statement should provide the following information, where applicable:

- Accession codes, unique identifiers, or web links for publicly available datasets
- A description of any restrictions on data availability
- For clinical datasets or third party data, please ensure that the statement adheres to our policy

All data will be available from the Cambridge Apollo Repository (https://doi.org/10.17863/CAM.96886)

Human research participants

Policy information about studies involving human research participants and Sex and Gender in Research.

Reporting on sex and gender	N/A
Population characteristics	N/A
Recruitment	NA
Ethics oversight	N/A

Note that full information on the approval of the study protocol must also be provided in the manuscript.

Field-specific reporting

Please select the one below that is the best fit for your research. If you are not sure, read the appropriate sections before making your selection.

☒ Life sciences ☐ Behavioural & social sciences ☐ Ecological, evolutionary & environmental sciences

For a reference copy of the document with all sections, see nature.com/documents/nr-reporting-summary-flat.pdf

Life sciences study design

All studies must disclose on these points even when the disclosure is negative.

Sample size	No statistical methods were used to predetermine sample size. Sample size was determined based on preliminary observations which determined how large sample size must be to obtain reproducible results.
Data exclusions	No data were excluded in this study.
Replication	Phenotyping experiments were replicated two or more times with similar results (numbers of individuals detailed in the respective figures/methods section).<br>Imaging data were obtained from multiple individual saplings for each assessed background (numbers detailed in each figure).<br>Biochemical analysis (PACE, Monosaccharide analysis, linkage analysis, Glycome profiling/ELISA, Lignin analysis) were performed in technical triplicates from pooled individuals (numbers detailed in each respective figure/methods section).<br>RAMAN analysis were obtained by averaging 24 spectra of 3 independent individuals per genetic background assessed.<br>Solid state NMR experiments were obtained from one biological replicate of 2 indepedent genetic lines versus their mock control.<br>Water vapor sorption was obtained on pooled individuals from the same line versus their DMSO control and replicated twice.<br>Thermoporosimetry experiments were carried out in triplicates from pooled individuals for the same genetic background (numbers detailed in the respective figure/methods section).<br>Biomass crystallinity was carried out on 8 independent individuals per genetic background assessed.<br>Tensile Properties were assessed on by performing 10 technical replicates on 10 independent individuals per genetic background assessed.<br>Green density was assessed on 10 independent individuals per genetic background assessed.<br>Cellulose microfibril angle (MFA) was assessed on 3 stem positions on 10 independent individuals per genetic background assessed.

	Saccharification and Simultaneous saccharification and ethanolic fermentation (SSF) were carried out as technical triplicates on pooled individuals (numbers detailed in the respective figure/methods sections).
Randomization	Individuals from different groups (genotypes) were grown simultaneously within each experiment repeat. All experiments compared genotypes/individuals grown simultaneously in order to avoid any batch effect.
Blinding	Blinding was not applied since experiments were carried out without previous expectations. To avoid any bias of genotype/treatment effect on data collection, all genotypes and conditions have been assessed simultaneously for each experiment.

Reporting for specific materials, systems and methods

We require information from authors about some types of materials, experimental systems and methods used in many studies. Here, indicate whether each material, system or method listed is relevant to your study. If you are not sure if a list item applies to your research, read the appropriate section before selecting a response.

Materials & experimental systems

n/a	Involved in the study
☐	☒ Antibodies
☒	☐ Eukaryotic cell lines
☒	☐ Palaeontology and archaeology
☒	☐ Animals and other organisms
☒	☐ Clinical data
☒	☐ Dual use research of concern

Methods

n/a	Involved in the study
☒	☐ ChIP-seq
☒	☐ Flow cytometry
☒	☐ MRI-based neuroimaging

Antibodies

Antibodies used

Callose was detected with an anti (1→3)-β-glucan primary antibody (Biosupplies, 400-2) and Alexa Fluor 488 anti-mouse IgG (ThermoFisher Scientific, catalog number A-11017).
Xylans were detected with LM10 and LM11 primary antibodies (PlantProbes).
glucuronoxylans were detected with LM28 primary antibodies (PlantProbes).
xyloglucans were detected with LM15, LM24 and LM25 primary antibodies (PlantProbes).
mannans were detected with LM21 and LM22 primary antibodies (PlantProbes).
Pectins were detected with LM7, LM18, LM19, LM20, JIM5, JIM7, LM5, LM26, LM6-M, LM13, and LM16 primary antibodies (PlantProbes).
Xylogalacturonans were detected with LM8 primary antibody (PlantProbes).
Xylosyl residues were detected with LM23 primary antibody (PlantProbes).
Arabinogalactan-protein (AGP) glycan were detected with LM2, LM14, JIM13, JIM15 and JIM16 primary antibodies (PlantProbes).
Extensins were detected with LM1 and JIM20 primary antibodies (PlantProbes).
No catalog nor clone numbers were provided by PlantProbes upon order.

Validation

Specificity of 400-2 anti (1→3)-β-glucan has been validated in Meikle et al. (1991). Planta 185: 1-8.
Specificity of LM10 and LM11 have been validated in McCartney et al. (2005) J. Histochem Cytochem 53, 543.
Specificity of LM28 has been validated in Cornuault et al. (2015) Planta 242,1321-1334.
Specificity of LM15 has been validated in Marcus et al. (2008) BMC Plant Biology 8, 60.
Specificity of LM24 and LM25 have been validated in Pedersen et al. (2012) J. Biol. Chem. 287, 39429-39438.
Specificity of LM21 and LM22 have been validated in Marcus et al. (2010) Plant Journal 64, 191-203.
Specificity of LM7 has been validated in Willats et al. (2001) J. Biol. Chem. 276, 19404-19413.
Specificity of LM18, LM19 and LM20 have been validated in Verhertbruggen et al. (2009) Carbohydr. Res. 344, 1858.
Specificity of JIM5 and JIM7 have been validated in Knox et al. (1990) Planta 181, 512-521.
Specificity of LM5 has been validated in Willats et al. (1999) Plant Journal 20, 619-628.
Specificity of LM26 has been validated in Torode et al. (2018) Plant Physiology 176, 1547-1558.
Specificity of LM6-M has been validated in Cornuault et al. (2018) BioRχiv /10.1101/161604.
Specificity of LM13 and LM16 have been validated in Verhertbruggen et al. (2009) Plant Journal 59, 413-425.
Specificity of LM8 has been validated in Willats et al. (2004) Planta 218, 673-681.
Specificity of LM23 has been validated in Manabe et al. (2011) Plant Physiology 155, 1068-1078.
Specificity of LM2 has been validated in Yates et al. (1996) Glycobiology 6, 131-139.
Specificity of LM14 has been validated in Moller et al. (2008) Glycoconjugate J. 25, 37-48.
Specificity of JIM13, JIM15 and JIM16 has been validated in Knox, et al. (1991) Plant Journal 1, 317-326.
