# Supplementary figures and images for: Ectopic callose deposition into woody biomass modulates the nano-architecture of macrofibrils
Source: Nat Plants. 2023 Sep 4;9(9):1530–46. doi: 10.1038/s41477-023-01459-0 (PMC10505557; doi:10.1038/s41477-023-01459-0)

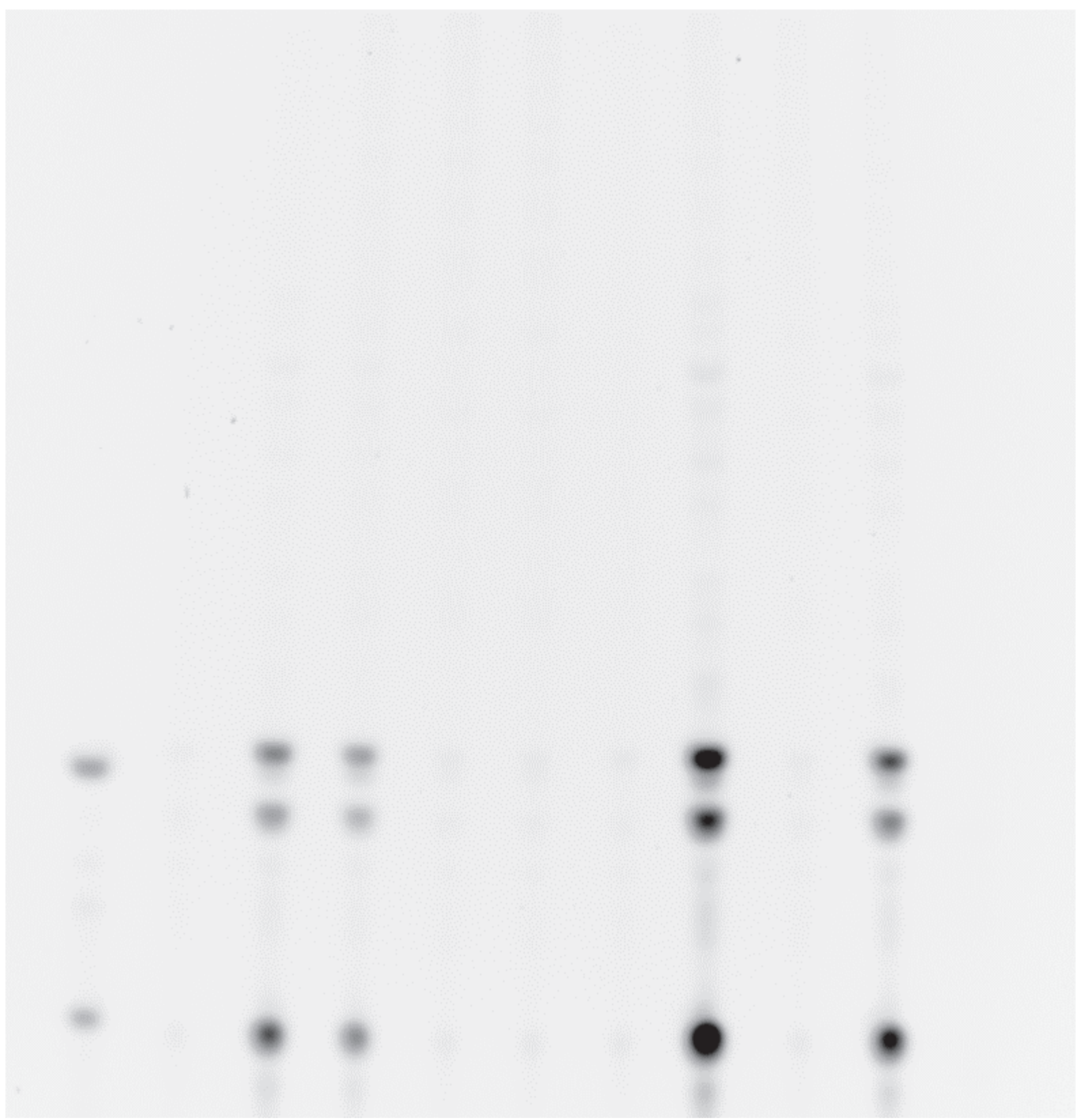

Supplement: Source Data Fig. 1 — Unprocessed PACE gel for Fig. 1m. [file 41477_2023_1459_MOESM3_ESM.pdf]

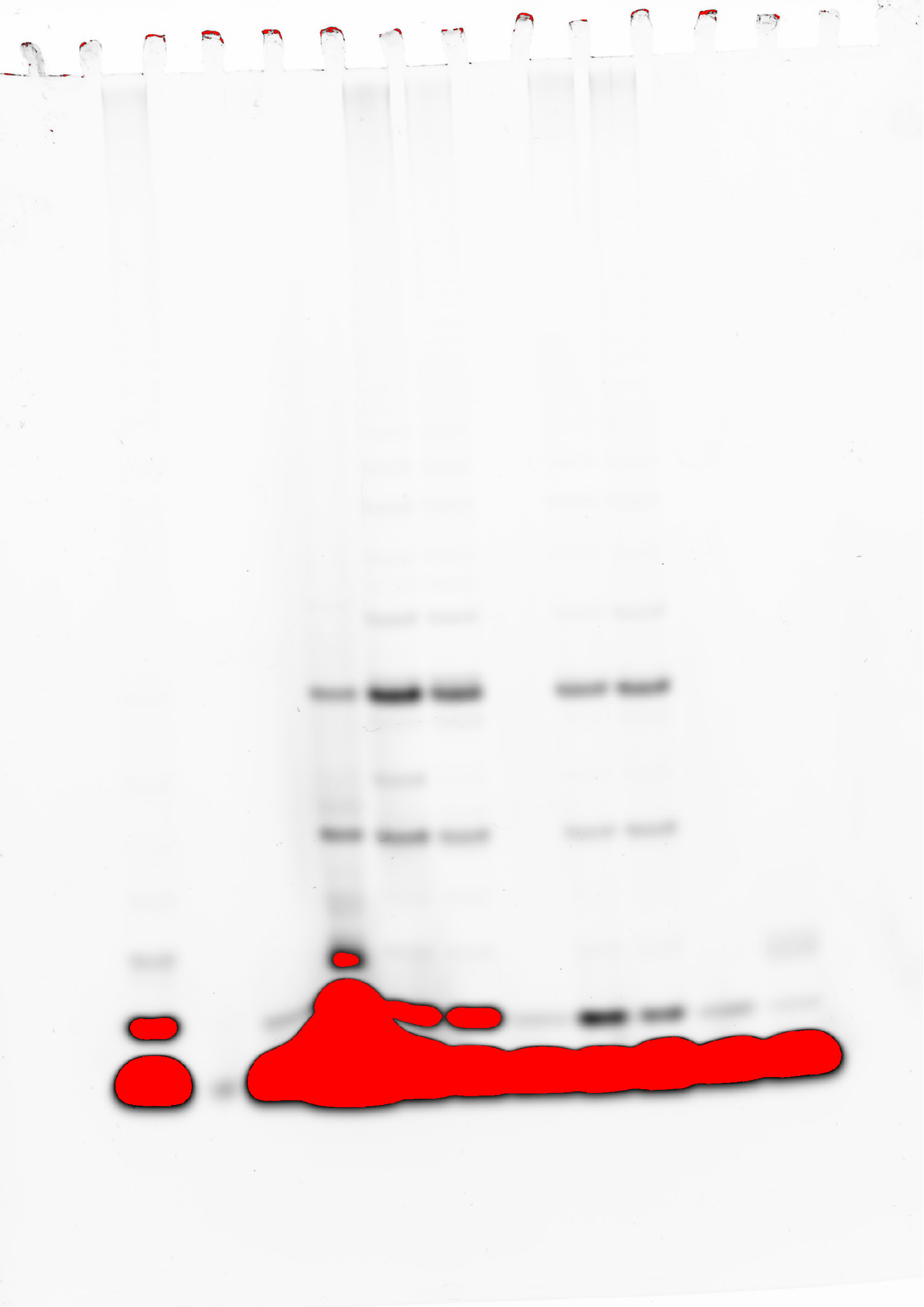

Supplement: Source Data Extended Data Fig. 3 — Unprocessed PACE gels for Extended Data Fig. 3d. [file 41477_2023_1459_MOESM4_ESM.pdf]
